# Dynamics of Expression Variability Contribute to Retention of Small-Scale vs. Whole-Genome Duplicates

**DOI:** 10.1093/gbe/evag077

**Published:** 2026-03-31

**Authors:** Haoran Cai, David L Des Marais

**Affiliations:** Department of Ecology and Evolutionary Biology, University of California, Los Angeles, CA 90095, USA; Department of Civil and Environmental Engineering, Massachusetts Institute of Technology, 15 Vassar St., Cambridge, MA 02139, USA; Department of Civil and Environmental Engineering, Massachusetts Institute of Technology, 15 Vassar St., Cambridge, MA 02139, USA

**Keywords:** gene duplication, variability, gene expression, evolvability

## Abstract

Genome analyses reveal that gene duplication in eukaryotes is pervasive, providing a primary source for the emergence of new genes. Nevertheless, the mechanisms influencing the probability of early duplicate retention and the emergence of functional biases,such as the enrichment of tandem duplicates in environmental responses, remain unclear. Here, to elucidate the mechanisms and factors determining gene retention, we study a frequently overlooked molecular feature—within-line expression variation, termed expression variability. We demonstrate that, on average, genes with duplicates exhibit higher expression variability than singletons. Furthermore, small-scale duplications (SSDs) and whole-genome duplications (WGDs) display contrasting functional outcomes and time-dependent profiles in expression variability. These findings suggest a potential overarching mechanism that facilitates gene expression divergence, functional gains of environmental responses, and duplicate retention following SSDs.

Significance Gene duplication is a central driver of evolutionary innovation, yet the mechanisms determining the retention and functional divergence of duplicated genes remain poorly resolved. Here, we found that expression variability—a molecular feature often overlooked—plays an important role in the differential retention of small-scale versus whole-genome duplicates. By uncovering distinct patterns of variability associated with duplication mode and age, our findings suggest that elevated expression variability promotes functional diversification, particularly in environmental response genes arising from small-scale duplications. This work points to the importance of accounting for gene expression dynamics in understanding the evolutionary outcomes following duplication events.

## Introduction

Gene duplication is recognized as a primary source of new genes and gene functions and has consequently been credited with great evolutionary importance (Ohno 1970; Des Marais and Rausher 2008). A single duplication event produces one additional copy, yielding two paralogous gene copies. By increasing redundancy, duplication can reduce selective constraint on copies and, in some cases, facilitate the evolution of novel gene functions that contribute to adaptation.

Duplicate gene copies can be generated through one of two broad mechanisms, namely small-scale or large-scale duplication events. Among the most dramatic forms of large-scale duplication is whole-genome duplication (WGD), which leads to a sudden expansion in genome size and, significantly, preserves the chromosomal context of most genes (Kuzmin et al. 2022). WGDs have likely occurred multiple times throughout 200 million years of angiosperm evolution (Vision et al. 2000; Van de Peer et al. 2017; Chen et al. 2024). Conversely, small-scale gene duplications (SSD), including proximal duplication, tandem duplication, and transposed duplication, result in duplication of a specific genomic region, often comprising a single gene (Zhang 2003; Wang et al. 2012b; Kuzmin et al. 2022). Plant genomes, on average, harbor duplicate copies for approximately 65% of annotated genes (Panchy et al. 2016). Most duplicates originate from WGD events and, accordingly, paleopolyploidization has been cited as an important source of evolutionary novelty for land plants (Panchy et al. 2016; Tiley et al. 2016). While WGD events lead to a sudden and massive expansion of the genome, they are often followed by extensive gene loss over evolutionary time (Lynch and Conery 2000).

Considerable evidence suggests that duplicates originating from WGD and SSD events differ in evolutionary rate, essentiality, and functional properties (Freeling 2009; Mattenberger et al. 2017; Qiao et al. 2019). For example, in the budding yeast *Saccharomyces cerevisiae*, SSD-derived duplicates more often show signatures consistent with neo-functionalization, whereas WGD-derived duplicates show patterns consistent with subfunctionalization when “function” is quantified using interaction partners in genetic-interaction networks (Fares et al. 2013). Network-based analyses in yeast likewise report higher functional similarity among WGD paralogs than among SSD paralogs (Hakes et al. 2007). In plants, both empirical surveys and evolutionary models predict that WGD and SSD events preferentially retain genes from different functional classes (Maere et al. 2005; Freeling 2009). Consistent with this, analyses in *Arabidopsis* and other plant genomes indicate that SSDs (especially tandem/proximal duplicates) are enriched for environmental and defense response genes, whereas WGD-retained duplicates are enriched for intracellular regulatory functions, including transcription factors, ribosomal proteins, and other core cellular processes (Seoighe and Gehring 2004; Rizzon et al. 2006; Hanada et al. 2008; Wang et al. 2012b; Qiao et al. 2019). However, the mechanisms driving these functional biases remain incompletely understood. As such, we remain uncertain whether genes retained following the different duplication sources are subjected to different evolutionary constraints, or whether the two classes of genes possess equivalent potential to generate novel function. The gene balance theory offers one plausible explanation for why WGD events predominantly retain transcription factors (Freeling 2009). It has been hypothesized that the more connected a gene product is—for example, as a component of stoichiometric protein complexes and/or as a hub in gene regulatory networks—the more likely the phenotype will change if dosage imbalance occurs. Therefore, the gene balance theory posits that if a tandem duplicate is highly connected, it is more prone to cause dosage imbalance and thus is very likely to be removed. Conversely, after WGD, loss of a highly connected duplicate would be more likely to cause dosage imbalance and deleterious effects and thus is more likely to be retained (Freeling 2009; Wang et al. 2012b).

Theoretical and empirical results show that most duplicated genes return to single copies after duplication because a functionally redundant duplicate will accumulate deleterious mutations and evolve into a pseudogene (Tautz 1992; Wagner 1998; Lynch and Conery 2000). Therefore, the earliest stage following duplication is key to understanding the role of gene and genome duplication in evolution (Innan and Kondrashov 2010). Classic models of duplicate retention include neofunctionalization (Ohno 1970; Kuzmin et al. 2022), subfunctionalization (e.g. the duplication-degeneration-complementation model, DDC) (Van Hoof 2005; Marshall et al. 2013), and escape from adaptive conflict (a special case of subfunctionalization)(Des Marais and Rausher 2008; Kuzmin et al. 2022).

Changes in gene expression can play an important role in the preservation of duplicated genes (Doebley and Lukens 1998; Francino 2005; Casneuf et al. 2006; Duarte et al. 2006; Ganko et al. 2007; Guschanski et al. 2017; Roux et al. 2017). One observation is that gene expression exhibits greater divergence for pairs originating from SSD as compared to WGD (Casneuf et al. 2006), which may arise from a higher likelihood of altering regulatory features during the process of SSD (e.g. if the duplication event does not faithfully replicate *cis*-regulatory elements) (Rogers et al. 2017). Although seemingly contradictory to such observations, WGD pairs, in fact, have a higher average number of *cis*-element differences between paralogs than do SSDs (Arsovski et al. 2015). Thus, a question remains as to whether changes in transcription contribute to the probability of gene retention and the subsequent functional biases observed in retained paralogs (Hallin and Landry 2019). Another relevant and intriguing question is whether variants affecting gene expression dosage experience the same degree of relaxed selection as do variants that affect protein coding sequences; it is generally accepted that functional redundancy of a protein-coding gene leads to relaxed selection constraints (Ohno 1970; Kondrashov et al. 2002). If this is also the case with transcriptional expression, it would naturally increase the “search space” of a new copy, and potentially facilitate gene retention via neofunctionalization.

Here, we study expression variability among gene duplicate pairs to test the hypothesis that biases in the functions of gene copies retained can be explained by changes in regulatory architecture immediately after duplication. Expression variability is defined as the variation of gene expression among genetically identical individuals in a controlled environment (Cortijo et al. 2019; Cortijo and Locke 2020). In multicellular organisms, such as plants, genetically identical or clonal individuals are used to assess variability, also referred to as inter-individual variability (Cortijo et al. 2019) or intra-genotypic variability (Metcalf and Ayroles 2020). In the context of gene duplication, a duplicate may change its genomic context, via transposition or incomplete duplication in which regulatory elements are reshuffled (Arsovski et al. 2015), thereby changing expression variability of the new copy as it becomes fixed through neofunctionalization. Here, we first show that duplicates arising from both SSDs and WGDs tend to exhibit elevated expression variability. Our subsequent analyses point to distinct mechanisms that could explain the increasing expression variability observed for SSDs and WGDs.

## Results

Following Cortijo et al. (2019), we first obtained normalized expression variability for each gene at each time point during a day. Gene expression data were acquired from 14 seedlings of the Col-0 wild type of *Arabidopsis thaliana*. The goal of normalization is to account for the technical bias—coupling between mean expression and variation around the mean, with the aim to compare expression variability across genes. Hereafter, we use median normalized expression variability among all time points to quantify a focal gene’s expression variability, termed normalized expression variability (NEV).

### Elevated Gene Expression Evolvability for Duplicates

**Fig. 1.**
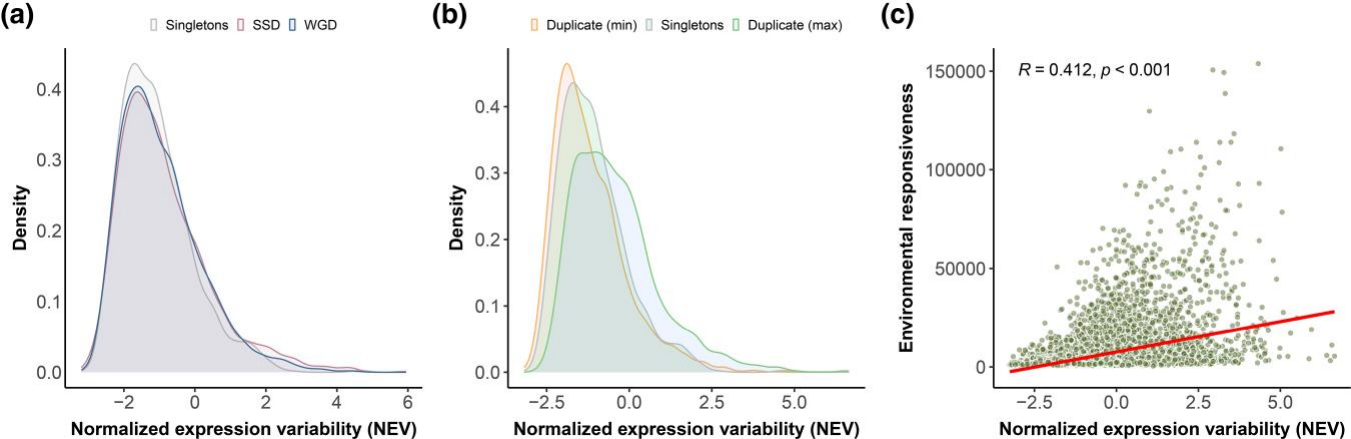
Overall properties of normalized expression variability (NEV) a). Distribution of NEV grouped by singletons (N=1241), genes arising from SSD (N=3366), and from WGD (N=3175). Genes born from whole genome duplications (WGDs) and small-scale duplications (SSDs) exhibit significantly higher NEV than singletons (Wilcoxon two-sided test adjusted P=0.047 and Cliff’s Delta effect size = 0.047 95% CI [0.010, 0.084] for WGDs vs singletons, Wilcoxon test adjusted P=0.00071 and Cliff’s Delta effect size = 0.070 95% CI [0.034, 0.106] for SSDs vs singletons, Wilcoxon test adjusted P=0.27 and Cliff’s Delta effect size = 0.024 95% CI [-0.004,0.052] between SSDs and WGDs). b). Distributions of NEV grouped by genes with lower NEVs within a paralog pair, singletons, and genes with higher NEVs within a paralog pair. N=1241 for singletons, N=4344 for each duplicate group (max and min) c). The plasticity of transcriptional responses (responsiveness) summarizes the ability of a gene’s transcript to vary in response to a wide range of environmental perturbations. In our case, we obtained an expression atlas and calculated responsiveness using expression data in seedlings and col-0 ecotype of *Arabidopsis* (Roberts and Josephs 2023). NEV correlates with plasticity of transcriptional responses. Numbers in the plot are the Pearson correlation coefficient and associated *P*-value

We first categorized genes into three groups using their duplication status acquired from Yang and Gaut (2011): singletons (i.e. no known duplicates in the *A. thaliana* genome), duplicates arising from WGD, and duplicates arising from SSD (Fig. 1a, Figs. S2, and S3). Despite the small between-group effect sizes (quantified by Cliff’s delta, *δ*, which measures the probability that a value from one group exceeds a value from another), the NEV distributions of both SSDs and WGDs are nonetheless significantly shifted relative to genes without duplicates (Fig. 1a; Fig. S3; adjusted P=0.0007 for SSDs and P=0.047 for WGDs). We detect no significant difference in NEV between SSDs and WGDs (Fig. 1a and Fig. S3, adjusted P=0.27). We also conducted similar analyses by stratifying the data based on specific features of genes (Fig. S1) and gene ages (Fig. S2). Further analysis reveals that this elevated expression variation is primarily driven by specific copies exhibiting higher NEVs, rather than uniformly high NEV across all duplicates (Fig. 1b).

### Expression Variability as an Evolvability Index for Gene Expression

We next ask whether expression variability is coupled with plasticity caused by macro-environmental variation, finding that genes with higher NEV tend to show higher sensitivity to environmental perturbations (Fig. 1c). Conversely, genes with lower NEV are more often insensitive to environmental perturbation. While one of the drivers of NEV is micro-environmental variation among individuals, such differences are small, random, and distinguishable from macro-environmental variation that characterizes systematic differences of environmental parameters in experimental settings (Masel and Siegal 2009). This distinction is apparent as indicated in Fig. 1c: higher NEV does not necessarily lead to higher sensitivity to environmental perturbations in an experiment, while a lower NEV constrains the environmental responsiveness of a gene (the apparently inaccessible top left area in the scatter plot of Fig. 1c).

Notably, such noise-plasticity coupling has been observed in single cell organisms, and has been interpreted as a constraint on the evolution of gene expression more broadly (Lehner 2010; Singh 2013; Chapal et al. 2019). We also observe a positive correlation between tissue specificity and NEV in two independent datasets, which were calculated from Arabidopsis Development Atlas (ADA) (Yang and Gaut 2011), and from the expression atlas (Roberts and Josephs 2023) (Fig. S4). One possibility is that tissue-specific genes exhibit higher variability because they require context-dependent regulation across different developmental stages or cellular environments.

Prior work on the effects of naturally occurring mutations on gene expression found that genes with greater sensitivity to mutation exhibit higher stochastic noise in gene expression and are also more sensitive to environmental perturbation (Landry et al. 2007). In single-cell organisms, stochastic noise describes the variation within a population of genetically identical cells under a single environment. Together with our study, these findings in yeast mutation accumulation lines imply that expression variability may play a role in the evolution of environmental responses more broadly. We thus hypothesize that elevated NEV facilitates the acquisition of novel environmental response functions, which may promote the probability of gene retention following duplication.

### Contrasting Functions among SSD and WGD Paralogs Despite Similar Levels of NEV

**Fig. 2.**
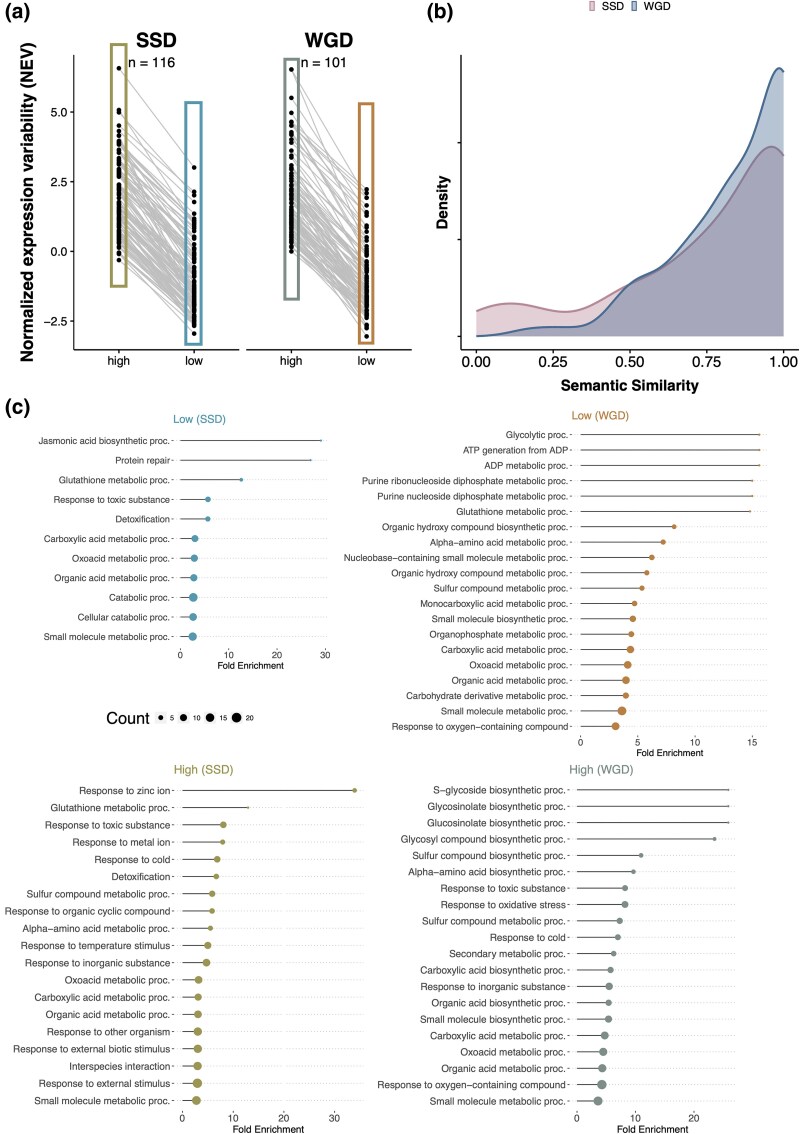
Contrasting GO enrichment results for SSDs and WGDs with NEV diverged gene pairs. a). Top 5% paralog pairs with strongest NEV divergence between two paralogs are shown as single lines for two duplication types (n=116, SSD; n=101, WGD). Repeated genes in each group are removed. b). Pairwise semantic similarity analyses are performed for each pair of paralog for SSDs and WGDs. R package *GOSemSim* (Yu et al. 2010) is used with parameters: measure = “Jiang”, combine = “BMA”, ont = “BP”. c). GO enrichment analyses are conducted using ShinyGo http://bioinformatics.sdstate.edu/go/ (Ge et al. 2020), for each group among the four in panel A separately (with corresponding colors). Lists of GO biological functions with FDR of 0.05 are shown. GO terms with less than 10 genes are excluded. Fold enrichment and gene count are shown with lollipop plot with the dot. Background set used for GO enrichment is all *Arabidopsis thaliana* protein-coding genes. Enrichment results of singletons with high and low NEVs are shown in Supplemental Tables 5 and 6.

We conducted GO enrichment and pairwise semantic similarity analyses on pairs of duplicated genes that show the greatest divergence in NEV between copies (the 5% of genes exhibiting the greatest divergence, comprising 116 SSD pairs and 101 WGD pairs, Fig. 2a and Fig. S7). The semantic similarity analyses indicate that diverged SSD pairs are more likely to exhibit more divergent functions between paralogs than pairs arising from WGDs (Fig. 2b, two additional similarity measures in Fig. S8, and analyses within each sub-type in Fig. S15). Next, we specifically tested whether the “high NEV” paralogs from each pair, as a group, exhibit different functional enrichments than the group of “low NEV” paralogs (Fig. 2c, Supplemental Tables 1–6). For SSDs, genes with higher NEV in a SSD paralog pair show distinct functions as compared to the lower group. In particular, paralogs in the SSD high group are enriched for functions in biotic and abiotic environmental responses such as responses to cold, zinc ion, fungi, and herbivores. Previous studies found that genes derived from SSD are more likely to be involved in response to environmental stimuli (Hanada et al. 2008). Here, we provide further evidence that, for SSDs, abiotic or biotic responses are specifically associated with increased NEVs. Notably, extant SSDs tend to be younger than WGD-derived paralogs based on $K_{s}$ distributions (Fig. S13, also see Wang et al. 2013; Rody et al. 2017), reinforcing our results that SSDs exhibit greater functional divergence despite shorter evolutionary time. In contrast, the GO enrichment results for low and high NEV groups of singletons (Supplemental Tables 5–6) and WGDs (Fig. 2c and Supplemental Table 3–4) do not exhibit similar patterns observed in SSDs. For example, both low and high NEV groups for WGDs are enriched with central metabolism and biosynthetic processes. These results suggest that the association between elevated NEVs and plastic responses is more pronounced with SSDs than WGDs.

We also observed bias in transcription factor retention between two duplication types. The list of TF in *Arabidopsis thaliana* is obtained from PlantTFDB (Jin et al. 2016). We assessed the number of transcription factor (TF) and non-TF paralog pairs in the SSD and WGD pools. (Cases in which a pair of paralogs comprise both a TF and a non-TF gene are rare and thus excluded.) SSDs including proximal, tandem, and transposed duplications have far fewer TF paralog pairs as compared to genes arising from WGD (Fig. S5, P-value=5.61e−96, chi-squared test), in line with previous results (Maere et al. 2005). Importantly, for tandem and proximal duplications, TF paralog pairs exhibit lower NEVs as compared to non-TF paralogs, and the distribution of NEV between TF and non-TF pairs is more divergent than other duplication types (Fig. S6). In contrast, we do not observe the same degree of separation between TF and non-TF NEV distributions for WGD pairs (Fig. S6). A possible interpretation is that SSDs are more likely to create local dosage imbalance and/or perturb *cis*-regulatory context, so TF duplicates arising via SSD may be selectively filtered unless they maintain relatively low NEV. In contrast, WGDs duplicate many interacting components simultaneously, better preserving relative dosage and maintaining chromosomal/regulatory context, which can reduce the selective penalty of retaining TF duplicates even when NEV is not exceptionally low.

### Distinct Mechanisms Leading to Elevated Expression Variability of WGDs and SSDs

Despite comparable elevation in NEV relative to singletons, WGDs and SSDs display distinct functional enrichment patterns among retained genes. We hypothesize that these differences stem from the timing and rate at which high expression variability is acquired. In principle, the *cis*- and *trans*-regulatory environments are likely conserved immediately after WGD events (Cisneros et al. 2026). Consequently, the increased NEV observed in WGD duplicates, relative to singletons, may result from the gradual accumulation of differences over extended evolutionary timescales. In contrast, the elevated NEV in SSDs may emerge during both the duplication process itself and the subsequent evolutionary time. Mechanisms such as translocation via transposable elements or reverse transcription can disrupt a paralog’s regulatory machinery, independently of selection or other long-term evolutionary pressures. As a result, the increased NEV observed in SSDs may reflect both the immediate disruptive effects of novel genomic contexts post-duplication and the preferential retention of duplicates with inherently higher NEVs, as those with lower variability may be more prone to early gene loss.

We thus tested whether SSDs exhibit a derived/ancestral bias in NEV. If new duplicates with elevated NEV are preferentially retained, derived copies should show higher NEV than their ancestral counterparts. To distinguish derived from ancestral copies, we used gene age estimates for 17,732 *Arabidopsis* genes inferred via phylostratigraphy (Domazet-Lošo et al. 2007; Arendsee et al. 2014). Importantly, we use this approach not to estimate duplication age, but to infer derived/ancestral status within each paralog pair: paralogs with higher clade numbers (younger) are more likely to be derived copies that translocated to novel genomic contexts, whereas paralogs with lower clade numbers (older) likely represent ancestral copies that remained in their original chromosomal location.

To validate this reasoning, we leveraged existing annotations of transposed duplicates (a subset of SSDs) in which parental and daughter copies were identified using synteny-based methods (MCScanX) (Wang et al. 2012a, 2013). Consistent with our interpretation, synteny-defined daughter loci tend to have higher clade numbers than parental loci (paired Wilcoxon test, $P=1.035\times 10^{-6}$; n=1373 pairs), supporting the use of phylostratigraphy to infer derived/ancestral status.

**Fig. 3.**
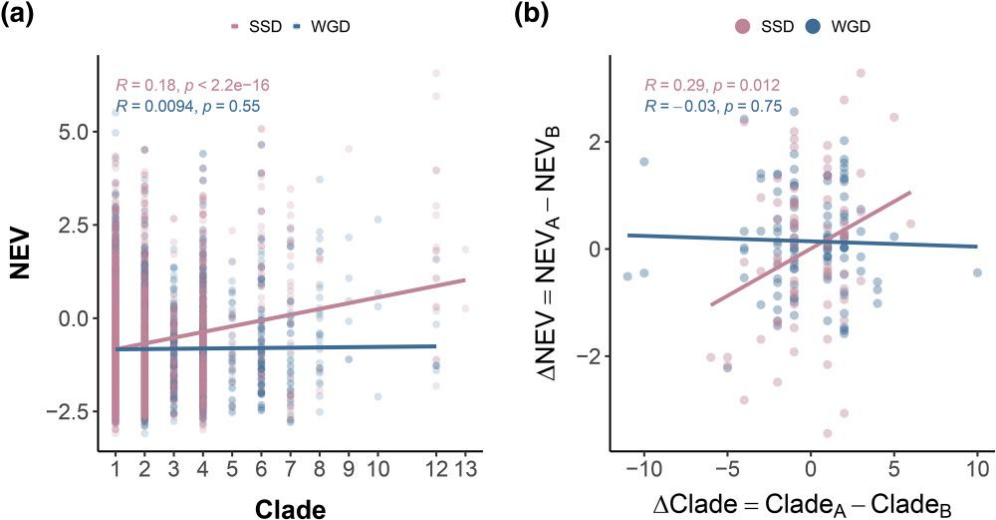
Paralog pairs arising from SSDs and WGDs exhibit distinct age-dependent patterns in NEVs. a) For duplicates arising from SSD, inferred derived genes (higher clade number) tend to exhibit higher NEV, whereas no significant pattern is observed for WGD duplicates. b) Within each paralog pair, we relate differences in inferred gene age to differences in NEV. We plot $\Delta \mathrm{Clade}={\mathrm{Clade}}_{A}-{\mathrm{Clade}}_{B}$ (higher clade number indicates an inferred derived gene) against $\Delta \mathrm{NEV}={\mathrm{NEV}}_{A}-{\mathrm{NEV}}_{B}$. Because *A* and *B* are assigned arbitrarily (i.e. no *a priori* parental/daughter designation), both ΔClade and ΔNEV can be positive or negative: ΔClade>0 indicates that gene *A* is inferred to be the derived copy relative to gene *B*, and ΔNEV>0 indicates that gene *A* has higher NEV than gene *B*. Paralog pairs with identical inferred clade are excluded (i.e. ΔClade=0). Gene age data were obtained from published data sets (Arendsee et al. 2014), comprising 17,732 Arabidopsis genes stratified into 15 clades, numbered from the oldest (stratum 1) to the most recent (stratum 15). Note that the phylostratigraphy approach is used here to infer derived/ancestral status, not duplication age.

Indeed, the positive trend for SSDs in Fig. 3a and b indicates that the derived copy in a paralog pair arising from SSD tends to have higher NEV than the ancestral copy. This derived/ancestral NEV asymmetry adds a new dimension to the well-documented pattern of asymmetric paralogue divergence—previously observed in sequence evolutionary rates, protein interactions, and tissue expression (Cisneros et al. 2026). By contrast, no such bias is observed for WGDs in both Fig. 3a and b. In addition, our analyses revealed consistently distinct temporal patterns for NEV divergence between SSDs and WGDs, when estimating duplication times with two independent methods (Figs. S10, S11, and S12). Though, within either duplication mode, the observed patterns were method-dependent and require further investigations.

Our findings support distinct models underlying elevated NEV in SSDs and WGDs. WGDs show no derived/ancestral bias, whereas SSDs exhibit a bias toward higher NEV for derived copies (Fig. 3b), driving the overall positive relationship between gene age and NEVs for all genes (Fig. S9). We also note that even after normalization, gene expression levels remain positively correlated with NEV (Fig. S14). To test whether the derived/ancestral bias can be driven by gene expression level, we repeated analyses by replacing NEV with gene expression level in Fig. 3B, the patterns for SSDs are opposite (Fig. S16), suggesting that the observed derived/ancestral bias is not driven by expression level effects.

## Discussion

Directly testing causality between NEV and retention probability is difficult with extant paralogs alone, because lost duplicates are not observed. Although the direction of causality between expression variability and gene retention cannot be determined conclusively, our analyses support a plausible filtering/ascertainment model to explain the elevated NEVs observed for SSDs. SSDs that arise from unequal crossing over during meiosis may not faithfully duplicate the complete *cis*-regulatory sequences, leading to mis-regulation as compared to the ancestral single copy gene (Rogers et al. 2017; Cisneros et al. 2026). SSDs that arise from translocation, e.g. via transposable elements or reverse transcription, may likewise find themselves in novel genomic contexts where local features such as chromatin structure (e.g. topological association domain), methylation, or *cis*-elements introduce novel expression phenotypes (Wang et al. 2012b). We emphasize that we are not proposing that translocation deterministically increases NEV; rather, translocation of a paralog to a novel genomic setting generates a range of regulatory outcomes. Among this distribution of regulatory outcomes, duplicates that happen to acquire higher NEV—potentially reflecting greater regulatory sensitivity or environmental responsiveness—may have a higher probability of retention, whereas low-NEV copies may be preferentially lost. However, our data do not allow us to cleanly separate “immediate” effects of relocation from gradual regulatory evolution over time. Therefore, we cannot rule out the possibility that the elevated NEVs observed for SSDs are acquired over evolutionary time rather than immediately upon duplication.

Still, in line with our model, we observe a bias towards higher NEV for derived copies in SSDs (Fig. 3b). Indeed, Qiao et al. (2019) synthesized duplication data across plant lineages and reported that proximal and tandem duplicates exhibited relatively high $K_{a}$/$K_{s}$ ratios but small $K_{s}$ values, possibly a signature of more rapid functional divergence as compared to other gene classes, and further supporting the hypothesis that positive selection plays an important role in the early stages following SSDs. In contrast to SSDs, we observe no significant derived/ancestral bias for WGDs in Fig. 3. We speculate that the overall elevated expression variability for WGD could potentially be due to relaxed selection arising from extra copies or positive selection over prolonged evolutionary time (Zhang et al. 2009).

As such, we posit that stress-responsive genes are more likely to be retained via SSD than via WGD. Conversely, the retention of transcription factors follows a different pattern. For transcription factors, dramatic regulatory and expression variability changes are likely deleterious as these proteins can be central and highly “connected” in gene regulatory networks and therefore subjected to selective pressure to minimize transcriptional noise (Puzović et al. 2023). Therefore, transcription factors are less likely to be retained following SSDs than WGDs. Our results propose a plausible explanation for reciprocal retention between tandem duplication and WGDs, potentially complementary to dosage balance theory and other existing models (Freeling 2009; Tasdighian et al. 2017).

The concept of inter-individual variability we study here is distinct from the expression noise in single-cell organisms, which is cell-to-cell heterogeneity in gene expression. Cell-to-cell heterogeneity in gene expression is due to the stochastic nature of the molecular interactions involved in (post)transcriptional regulation (Elowitz et al. 2002; Pedraza and van Oudenaarden 2005; Raser and O’shea 2005; Das Neves et al. 2010; Battich et al. 2015). In addition to cell-to-cell heterogeneity in gene expression, inter-individual expression variability can arise from micro-environmental variation among individuals (during development) and stochastic variation in cell-type compositions among individuals (bulk RNA-seq experiments measure total gene expression from heterogeneous tissues). Micro-environmental variation, which leads to cell-to-cell heterogeneity and inter-individual variability, is distinct from macro-environmental variation, which regulates stress responses and phenotypic plasticity (Hall et al. 2007; Siegal and Leu 2014; Cortijo et al. 2019; Paaby and Testa 2021). In experimental settings, macro-environmental variation refers to systematic environmental differences deliberately introduced by the experimenter, whereas micro-environmental variation encompasses mild, non-directional random fluctuations (Masel and Siegal 2009).

Low genetic variation is often interpreted as an evolutionary constraint. However, in many systems, within-line phenotypic variability and “noise” covary with both genetic variation and environmental plasticity—the so-called congruence hypothesis (Wagner et al. 1997; Gibson and Wagner 2000; Hansen 2006). One explanation is that diverse perturbations (genetic, micro-environmental, or macro-environmental) can act through shared regulatory and developmental dynamics, such that variation produced under one source of perturbation can be aligned with variation produced under others (Alberch 1982, 1989; Landry et al. 2007; Nuño de la Rosa and Müller 2024). In this framework, we use the term “expression-level evolvability” to denote a gene’s capacity to access alternative expression states when regulatory inputs change. Our results suggest that NEV captures an empirically measurable component of this capacity: genes with higher inter-individual variability also tend to be more environmentally responsive. Thus, we propose that expression variability can serve as a practical proxy for expression-level evolvability (Rocabert et al. 2020; Draghi and Ogbunugafor 2023; Cai et al. 2025), with potential relevance for understanding how duplicates acquire novel functions and diverge in regulation.

## Materials and Methods

### Normalized Expression Variability

Gene expression data was obtained from Cortijo et al. (2019). Briefly, RNA-sequencing of individual seedlings of *A. thaliana* (Col-0 accession) were used to calculate by-gene inter-individual expression variability. For a given gene and time point sampled, the square coefficient of variation $\left(CV^{2}=\frac{var}{mean^{2}}\right)$ is first calculated across the 14 seedlings. To account for the coupling between the mean gene expression level and $CV^{2}$ and to allow for comparisons among genes, normalized expression variability (NEV) for each gene at a given time point is calculated as (Cortijo et al. 2019; Brennecke et al. 2013):


$$NEV=log_{2}\frac{CV^{2}}{\hat{CV^{2}}(\mu )}$$

where $\hat{CV^{2}}(\mu )$ is the expected $CV^{2}$ for genes with similar mean expression, estimated by fitting a trend line to the mean–variance relationship. Under this definition, NEV<0 indicates that a gene’s expression variability is lower than expected for genes with similar mean expression, whereas NEV>0 indicates higher-than-expected variability. This normalization procedure was originally developed to remove technical bias in RNA-seq measurements (Brennecke et al. 2013). We then used the median NEV across time points as each gene’s summarized NEV. NEV thus reflects a gene’s typical inter-individual expression variability, rather than within-individual temporal fluctuation. We note that a residual positive correlation between NEV and mean expression level persists after normalization (Fig. S14), which may reflect genuine biological coupling between expression level and expression variability as well as remaining technical artifacts.

### Calculation of Environmental Responsiveness from Expression Atlas

We used a large gene expression atlas (Roberts and Josephs 2023), encompassing a range of experimental conditions and tissues, to characterize the plastic expression responses of individual genes. To calculate environmental responsiveness, we focused on the “Col-0” ecotype and ‘seedling’ tissue across environmental treatments (e.g. cold exposure, day length, hormonal treatment) and developmental stages in the atlas. Responsiveness was quantified as the sum of log2 ratios between expression in a treatment and a baseline condition:


$$\Sigma \log_{2}\;\frac{expr}{expr_{baseline}}$$

### Tissue Specificity

Tissue specificity of individual genes were obtained from Yang and Gaut (2011), which were calculated from Arabidopsis Development Atlas (ADA), and from the expression atlas (Roberts and Josephs 2023). The expression level of a gene was estimated by the average value of all 79 samples in Roberts and Josephs (2023). The tissue specificity was measured with the index *τ* (Yanai et al. 2005):


$$\tau =\frac{\sum_{\;j=1}^{n}\left(1-\frac{\log_{2}\;S_{i,j}}{\log_{2}\;S_{i,\max}}\right)}{n-1}$$

where *n* is the number of tissues, $S_{i,j}$ is the expression of gene *i* in tissue *j* , and $S_{i,\max}$ is the highest expression of gene *i* across *n* tissues. The index *τ* ranges from 0 to 1, with a higher value indicating a higher specificity. If a gene is expressed in only one tissue, *τ* approaches 1.

### Paralog Pairs

The designations of genes as duplicates or singletons were acquired from Yang and Gaut (2011) according to the assignments of Blanc et al. (2003). Paralogs and their corresponding duplication modes were obtained from Coate et al. (2020) and Wang et al. (2013). $k_{s}$ were similarly obtained from Coate et al. (2020), where alignments of protein sequences of duplicate genes were produced using ClustalW (Thompson et al. 1994).

## Supplementary Material

evag077_Supplementary_Data

## Data Availability

Data and code have been deposited in GitHub (https://github.com/haorancai/expr_variability).

## References

[evag077-B1] Alberch P . Developmental constraints in evolutionary processes. In: Evolution and Development: Report of the Dahlem Workshop on Evolution and Development Berlin 1981, May 10–15. Springer; 1982. p. 313–332.

[evag077-B2] Alberch P . The logic of monsters: evidence for internal constraint in development and evolution. Geobios. 1989:22:21–57. 10.1016/S0016-6995(89)80006-3.

[evag077-B3] Arendsee ZW, Li L, Wurtele ES. Coming of age: orphan genes in plants. Trends Plant Sci. 2014:19:698–708. 10.1016/j.tplants.2014.07.003. 25151064

[evag077-B4] Arsovski AA, Pradinuk J, Guo XQ, Wang S, Adams KL. Evolution of cis-regulatory elements and regulatory networks in duplicated genes of arabidopsis. Plant Physiol. 2015:169:2982–2991. 10.1104/pp.15.00717. 26474639 PMC4677880

[evag077-B5] Battich N, Stoeger T, Pelkmans L. Control of transcript variability in single mammalian cells. Cell. 2015:163:1596–1610. 10.1016/j.cell.2015.11.018. 26687353

[evag077-B6] Blanc G, Hokamp K, Wolfe KH. A recent polyploidy superimposed on older large-scale duplications in the arabidopsis genome. Genome Res. 2003:13:137–144. 10.1101/gr.751803. 12566392 PMC420368

[evag077-B7] Brennecke P et al Accounting for technical noise in single-cell RNA-seq experiments. Nat Methods. 2013:10:1093–1095. 10.1038/nmeth.2645. 24056876

[evag077-B8] Cai H, Melo D, Des Marais D. Disentangling variational bias: the roles of development, mutation and selection. Trends Genet. 2025:41:23–32. 39443198 10.1016/j.tig.2024.09.008

[evag077-B9] Casneuf T, De Bodt S, Raes J, Maere S, Van de Peer Y. Nonrandom divergence of gene expression following gene and genome duplications in the flowering plant arabidopsis thaliana. Genome Biol. 2006:7:1–11. 10.1186/gb-2006-7-2-r13. PMC1431724 16507168

[evag077-B10] Chapal M, Mintzer S, Brodsky S, Carmi M, Barkai N. Resolving noise–control conflict by gene duplication. PLoS Biol. 2019:17:e3000289. 10.1371/journal.pbio.3000289. 31756183 PMC6874299

[evag077-B11] Chen H, Almeida-Silva F, Logghe G, Bonte D, Van de Peer Y. 2024. The rise of polyploids during environmental catastrophes [preprint]. bioRxiv. 10.1101/2024.11.22.624806. 42105759

[evag077-B12] Cisneros AF et al Evolutionary causes and consequences of gene duplication. Nat Rev Genet. 2026:1–18. 10.1038/s41576-025-00917-z. 41699381

[evag077-B13] Coate JE, Farmer AD, Schiefelbein JW, Doyle JJ. Expression partitioning of duplicate genes at single cell resolution in arabidopsis roots. Front Genet. 2020:11:596150. 10.3389/fgene.2020.596150. 33240334 PMC7670048

[evag077-B14] Cortijo S, Aydin Z, Ahnert S, Locke JC. Widespread inter-individual gene expression variability in arabidopsis thaliana. Mol Syst Biol. 2019:15:e8591. 10.15252/msb.20188591. 30679203 PMC6346214

[evag077-B15] Cortijo S, Locke JC. Does gene expression noise play a functional role in plants? Trends Plant Sci. 2020:25:1041–1051. 10.1016/j.tplants.2020.04.017. 32467064

[evag077-B16] Das Neves RP et al Connecting variability in global transcription rate to mitochondrial variability. PLoS Biol. 2010:8:e1000560. 10.1371/journal.pbio.1000560. 21179497 PMC3001896

[evag077-B17] Des Marais DL, Rausher MD. Escape from adaptive conflict after duplication in an anthocyanin pathway gene. Nature. 2008:454:762–765. 10.1038/nature07092. 18594508

[evag077-B18] Doebley J, Lukens L. Transcriptional regulators and the evolution of plant form. Plant Cell. 1998:10:1075–1082. 10.1105/tpc.10.7.1075. 9668128 PMC1464652

[evag077-B19] Domazet-Lošo T, Brajković J, Tautz D. A phylostratigraphy approach to uncover the genomic history of major adaptations in metazoan lineages. Trends Genet. 2007:23:533–539. 10.1016/j.tig.2007.08.014. 18029048

[evag077-B20] Draghi JA, Ogbunugafor CB. Exploring the expanse between theoretical questions and experimental approaches in the modern study of evolvability. J Exp Zool B Mol Dev Evol. 2023:340:8–17. 10.1002/jez.b.23134. 35451559 PMC10083935

[evag077-B21] Duarte JM et al Expression pattern shifts following duplication indicative of subfunctionalization and neofunctionalization in regulatory genes of arabidopsis. Mol Biol Evol. 2006:23:469–478. 10.1093/molbev/msj051. 16280546

[evag077-B22] Elowitz MB, Levine AJ, Siggia ED, Swain PS. Stochastic gene expression in a single cell. Science. 2002:297:1183–1186. 10.1126/science.1070919. 12183631

[evag077-B23] Fares MA, Keane OM, Toft C, Carretero-Paulet L, Jones GW. The roles of whole-genome and small-scale duplications in the functional specialization of saccharomyces cerevisiae genes. PLoS Genet. 2013:9:e1003176. 10.1371/journal.pgen.1003176. 23300483 PMC3536658

[evag077-B24] Francino MP . An adaptive radiation model for the origin of new gene functions. Nat Genet. 2005:37:573–578. 10.1038/ng1579. 15920518

[evag077-B25] Freeling M . Bias in plant gene content following different sorts of duplication: tandem, whole-genome, segmental, or by transposition. Annu Rev Plant Biol. 2009:60:433–453. 10.1146/annurev.arplant.043008.092122. 19575588

[evag077-B26] Ganko EW, Meyers BC, Vision TJ. Divergence in expression between duplicated genes in arabidopsis. Mol Biol Evol. 2007:24:2298–2309. 10.1093/molbev/msm158. 17670808

[evag077-B27] Ge SX, Jung D, Yao R. Shinygo: a graphical gene-set enrichment tool for animals and plants. Bioinformatics. 2020:36:2628–2629. 10.1093/bioinformatics/btz931. 31882993 PMC7178415

[evag077-B28] Gibson G, Wagner G. Canalization in evolutionary genetics: a stabilizing theory? Bioessays. 2000:22:372–380. 10.1002/(SICI)1521-1878(200004)22:4¡372::AID-BIES7¿3.0.CO;2-J. 10723034

[evag077-B29] Guschanski K, Warnefors M, Kaessmann H. The evolution of duplicate gene expression in mammalian organs. Genome Res. 2017:27:1461–1474. 10.1101/gr.215566.116. 28743766 PMC5580707

[evag077-B30] Hakes L, Pinney JW, Lovell SC, Oliver SG, Robertson DL. All duplicates are not equal: the difference between small-scale and genome duplication. Genome Biol. 2007:8:1–13. 10.1186/gb-2007-8-10-r209. PMC2246283 17916239

[evag077-B31] Hall MC, Dworkin I, Ungerer MC, Purugganan M. Genetics of microenvironmental canalization in arabidopsis thaliana. Proc Natl Acad Sci U S A. 2007:104:13717–13722. 10.1073/pnas.0701936104. 17698961 PMC1959448

[evag077-B32] Hallin J, Landry CR. Regulation plays a multifaceted role in the retention of gene duplicates. PLoS Biol. 2019:17:e3000519. 10.1371/journal.pbio.3000519. 31756186 PMC6874296

[evag077-B33] Hanada K, Zou C, Lehti-Shiu MD, Shinozaki K, Shiu S-H. Importance of lineage-specific expansion of plant tandem duplicates in the adaptive response to environmental stimuli. Plant Physiol. 2008:148:993–1003. 10.1104/pp.108.122457. 18715958 PMC2556807

[evag077-B34] Hansen TF . The evolution of genetic architecture. Annu Rev Ecol Evol Syst. 2006:37:123–157. 10.1146/annurev.ecolsys.37.091305.110224.

[evag077-B35] Innan H, Kondrashov F. The evolution of gene duplications: classifying and distinguishing between models. Nat Rev Genet. 2010:11:97–108. 10.1038/nrg2689. 20051986

[evag077-B36] Jin J et al Planttfdb 4.0: toward a central hub for transcription factors and regulatory interactions in plants. Nucleic Acids Res. 2016:45:D1040–D1045. 10.1093/nar/gkw982 27924042 PMC5210657

[evag077-B37] Kondrashov FA, Rogozin IB, Wolf YI, Koonin EV. Selection in the evolution of gene duplications. Genome Biol. 2002:3:1–9. 10.1186/gb-2002-3-2-research0008. PMC65685 11864370

[evag077-B38] Kuzmin E, Taylor JS, Boone C. Retention of duplicated genes in evolution. Trends Genet. 2022:38:59–72. 10.1016/j.tig.2021.06.016. 34294428 PMC8678172

[evag077-B39] Landry CR, Lemos B, Rifkin SA, Dickinson W, Hartl DL. Genetic properties influencing the evolvability of gene expression. Science. 2007:317:118–121. 10.1126/science.1140247. 17525304

[evag077-B40] Lehner B . Conflict between noise and plasticity in yeast. PLoS Genet. 2010:6:e1001185. 10.1371/journal.pgen.1001185. 21079670 PMC2973811

[evag077-B41] Lynch M, Conery JS. The evolutionary fate and consequences of duplicate genes. Science. 2000:290:1151–1155. 10.1126/science.290.5494.1151. 11073452

[evag077-B42] Maere S et al Modeling gene and genome duplications in eukaryotes. Proc Natl Acad Sci U S A. 2005:102:5454–5459. 10.1073/pnas.0501102102. 15800040 PMC556253

[evag077-B43] Marshall AN, Montealegre MC, Jiménez-López C, Lorenz MC, van Hoof A. Alternative splicing and subfunctionalization generates functional diversity in fungal proteomes. PLoS Genet. 2013:9:e1003376. 10.1371/journal.pgen.1003376. 23516382 PMC3597508

[evag077-B44] Masel J, Siegal ML. Robustness: mechanisms and consequences. Trends Genet. 2009:25:395–403. 10.1016/j.tig.2009.07.005. 19717203 PMC2770586

[evag077-B45] Mattenberger F, Sabater-Muñoz B, Toft C, Fares MA. The phenotypic plasticity of duplicated genes in saccharomyces cerevisiae and the origin of adaptations. G3 (Bethesda). 2017:7:63–75. 10.1534/g3.116.035329. 27799339 PMC5217124

[evag077-B46] Metcalf CJE, Ayroles JF. Why does intragenotypic variance persist? In: Dobson A, Tilman D, Holt R, editors. Unsolved problems in ecology. Princeton University Press; 2020. p. 43–54. 10.1515/9780691195322-006.

[evag077-B47] Nuño de la Rosa L, Müller GB. The legacy and evolvability of pere alberch’s ideas. Interface Focus. 2024:14:20240011. 10.1098/rsfs.2024.0011. 39464645 PMC11503022

[evag077-B48] Ohno S . Evolution by gene duplication. Springer-Verlag. 1970.

[evag077-B49] Paaby AB, Testa ND. Developmental plasticity and evolution. In: Evolutionary developmental biology. Springer; 2021. p. 1073–1086. 10.1007/978-3-319-32979-6_110.

[evag077-B50] Panchy N, Lehti-Shiu M, Shiu S-H. Evolution of gene duplication in plants. Plant Physiol. 2016:171:2294–2316. 10.1104/pp.16.00523. 27288366 PMC4972278

[evag077-B51] Pedraza JM, van Oudenaarden A. Noise propagation in gene networks. Science. 2005:307:1965–1969. 10.1126/science.1109090. 15790857

[evag077-B52] Puzović N, Madaan T, Dutheil JY. Being noisy in a crowd: differential selective pressure on gene expression noise in model gene regulatory networks. PLoS Comput Biol. 2023:19:e1010982. 10.1371/journal.pcbi.1010982. 37079488 PMC10118199

[evag077-B53] Qiao X et al Gene duplication and evolution in recurring polyploidization–diploidization cycles in plants. Genome Biol. 2019:20:1–23. 10.1186/s13059-018-1612-0. 30791939 PMC6383267

[evag077-B54] Raser JM, O’shea EK. Noise in gene expression: origins, consequences, and control. Science. 2005:309:2010–2013. 10.1126/science.1105891. 16179466 PMC1360161

[evag077-B55] Rizzon C, Ponger L, Gaut BS. Striking similarities in the genomic distribution of tandemly arrayed genes in arabidopsis and rice. PLoS Comput Biol. 2006:2:e115. 10.1371/journal.pcbi.0020115. 16948529 PMC1557586

[evag077-B56] Roberts M, Josephs EB. Weaker selection on genes with treatment-specific expression consistent with a limit on plasticity evolution in arabidopsis thaliana. Genetics. 2023:224:iyad074. 10.1093/genetics/iyad074. 37094602 PMC10484170

[evag077-B57] Rocabert C, Beslon G, Knibbe C, Bernard S. Phenotypic noise and the cost of complexity. Evolution. 2020:74:2221–2237. 10.1111/evo.14083. 32820537

[evag077-B58] Rody HV, Baute GJ, Rieseberg LH, Oliveira LO. Both mechanism and age of duplications contribute to biased gene retention patterns in plants. BMC Genomics. 2017:18:46. 10.1186/s12864-016-3423-6. 28061859 PMC5219802

[evag077-B59] Rogers RL, Shao L, Thornton KR. Tandem duplications lead to novel expression patterns through exon shuffling in drosophila yakuba. PLoS Genet. 2017:13:e1006795. 10.1371/journal.pgen.1006795. 28531189 PMC5460883

[evag077-B60] Roux J, Liu J, Robinson-Rechavi M. Selective constraints on coding sequences of nervous system genes are a major determinant of duplicate gene retention in vertebrates. Mol Biol Evol. 2017:34:2773–2791. 10.1093/molbev/msx199. 28981708 PMC5850798

[evag077-B61] Seoighe C, Gehring C. Genome duplication led to highly selective expansion of the arabidopsis thaliana proteome. Trends Genet. 2004:20:461–464. 10.1016/j.tig.2004.07.008. 15363896

[evag077-B62] Siegal ML, Leu J-Y. On the nature and evolutionary impact of phenotypic robustness mechanisms. Annu Rev Ecol Evol Syst. 2014:45:495–517. 10.1146/annurev-ecolsys-120213-091705. PMC4448758 26034410

[evag077-B63] Singh GP . Coupling between noise and plasticity in e. coli. G3 (Bethesda). 2013:3:2115–2120. 10.1534/g3.113.008540. 24122054 PMC3852374

[evag077-B64] Tasdighian S et al Reciprocally retained genes in the angiosperm lineage show the hallmarks of dosage balance sensitivity. Plant Cell. 2017:29:2766–2785. 10.1105/tpc.17.00313. 29061868 PMC5728127

[evag077-B65] Tautz D . Problems and paradigms: redundancies, development and the flow of information. Bioessays. 1992:14:263–266. 10.1002/bies.950140410. 1596275

[evag077-B66] Thompson JD, Higgins DG, Gibson TJ. Clustal w: improving the sensitivity of progressive multiple sequence alignment through sequence weighting, position-specific gap penalties and weight matrix choice. Nucleic Acids Res. 1994:22:4673–4680. 10.1093/nar/22.22.4673. 7984417 PMC308517

[evag077-B67] Tiley GP, Ane C, Burleigh JG. Evaluating and characterizing ancient whole-genome duplications in plants with gene count data. Genome Biol Evol. 2016:8:1023–1037. 10.1093/gbe/evw058. 26988251 PMC4860690

[evag077-B68] Van de Peer Y, Mizrachi E, Marchal K. The evolutionary significance of polyploidy. Nat Rev Genet. 2017:18:411–424. 10.1038/nrg.2017.26. 28502977

[evag077-B69] Van Hoof A . Conserved functions of yeast genes support the duplication, degeneration and complementation model for gene duplication. Genetics. 2005:171:1455–1461. 10.1534/genetics.105.044057. 15965245 PMC1456075

[evag077-B70] Vision TJ, Brown DG, Tanksley SD. The origins of genomic duplications in arabidopsis. Science. 2000:290:2114–2117. 10.1126/science.290.5499.2114. 11118139

[evag077-B71] Wagner A . The fate of duplicated genes: loss or new function? Bioessays. 1998:20:785 788. 10.1002/(SICI)1521-1878(199810)20:10¡785::AID-BIES2¿3.0.CO;2-M. 10200118

[evag077-B72] Wagner GP, Booth G, Bagheri-Chaichian H. A population genetic theory of canalization. Evolution. 1997:51:329–347. 10.1111/j.1558-5646.1997.tb02420.x. 28565347

[evag077-B74] Wang Y, Tan X, Paterson AH. Different patterns of gene structure divergence following gene duplication in arabidopsis. BMC Genomics. 2013:14:1–9. 10.1186/1471-2164-14-1. 24063813 PMC3848917

[evag077-B75] Wang Y, Wang X, Paterson AH. Genome and gene duplications and gene expression divergence: a view from plants. Ann N Y Acad Sci. 2012b:1256:1–14. 10.1111/j.1749-6632.2011.06384.x. 22257007

[evag077-B73] Wang Y et al Mcscanx: a toolkit for detection and evolutionary analysis of gene synteny and collinearity. Nucleic Acids Res. 2012a:40:e49. 10.1093/nar/gkr1293. 22217600 PMC3326336

[evag077-B76] Yanai I et al Genome-wide midrange transcription profiles reveal expression level relationships in human tissue specification. Bioinformatics. 2005:21:650–659. 10.1093/bioinformatics/bti042. 15388519

[evag077-B77] Yang L, Gaut BS. Factors that contribute to variation in evolutionary rate among arabidopsis genes. Mol Biol Evol. 2011:28:2359–2369. 10.1093/molbev/msr058. 21389272

[evag077-B78] Yu G et al Gosemsim: an R package for measuring semantic similarity among go terms and gene products. Bioinformatics. 2010:26:976–978. 10.1093/bioinformatics/btq064. 20179076

[evag077-B79] Zhang J . Evolution by gene duplication: an update. Trends Ecol Evol. 2003:18:292–298. 10.1016/S0169-5347(03)00033-8.

[evag077-B80] Zhang Z, Qian W, Zhang J. Positive selection for elevated gene expression noise in yeast. Mol Syst Biol. 2009:5:299. 10.1038/msb.2009.58. 19690568 PMC2736655

