# Supplementary material for: Dynamics of Expression Variability Contribute to Retention of Small-Scale vs. Whole-Genome Duplicates
Source: Genome Biol Evol. 2026 Mar 31;18(4):evag077. doi: 10.1093/gbe/evag077 (PMC13069573; doi:10.1093/gbe/evag077)
Supplement: evag077_Supplementary_Data [file evag077_supplementary_data.zip › 12121sMain_SI.pdf]

Supplemental Figures

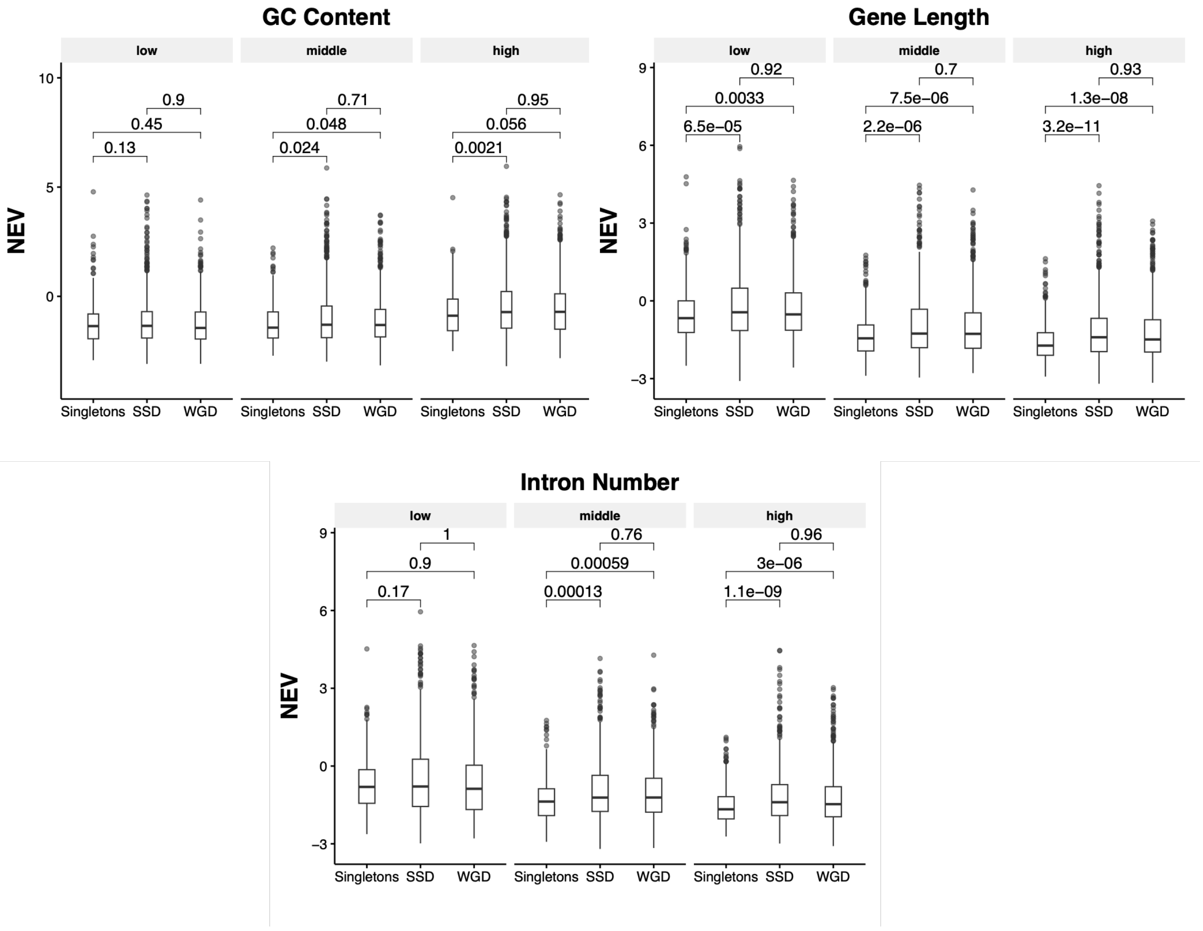

Figure S1: Distribution of normalized expression variability (NEV) grouped by genomic features (GC content, gene length, intron number) and duplication types. Numbers indicate pairwise p-values from Wilcox test among singletons, duplicates arising from SSDs, duplicates arising from WGDs.

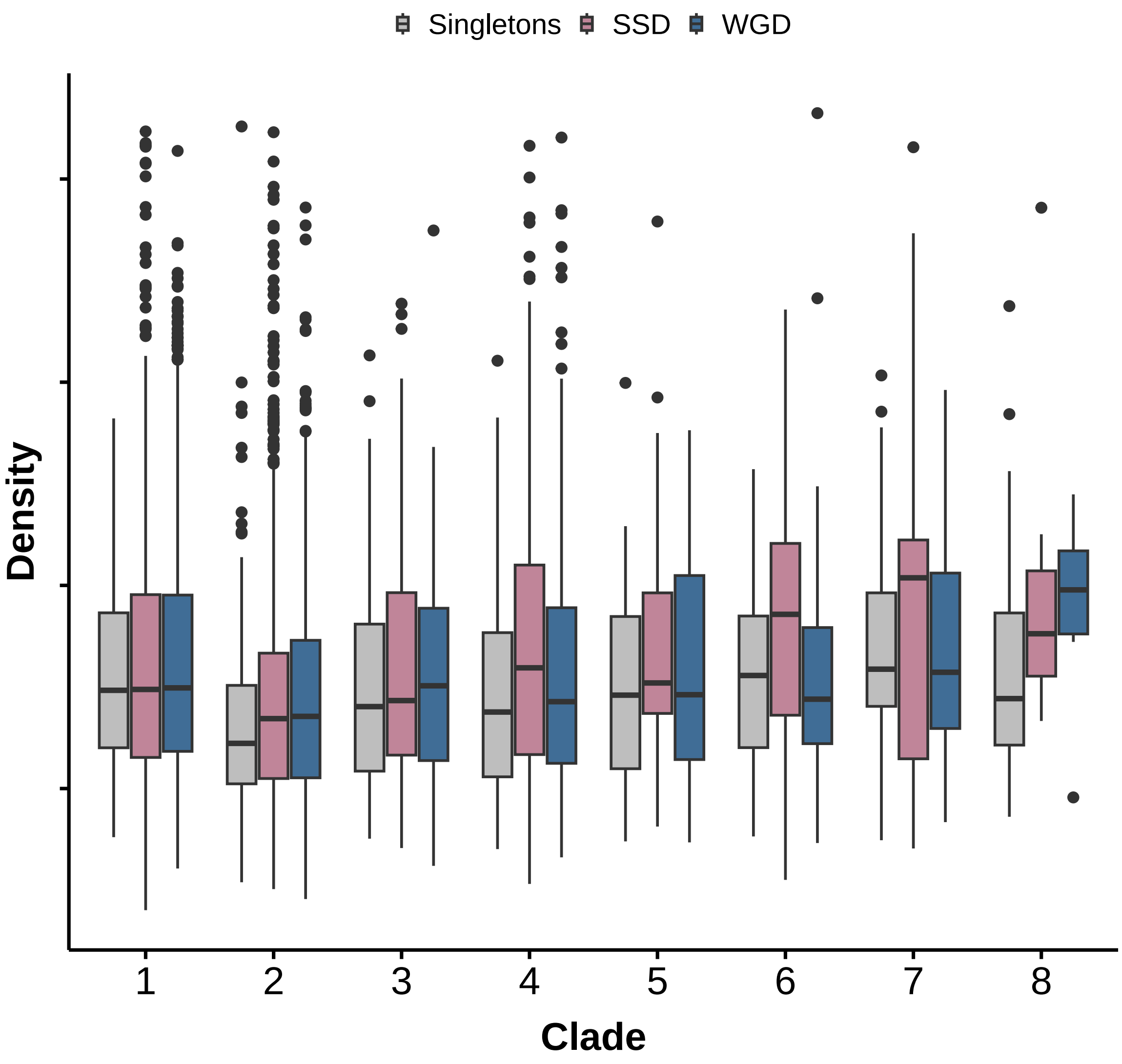

Figure S2: Comparisons of normalized expression variability (NEV) among singletons, SSDs, and WGDs grouped by gene age. Higher clade number represents younger genes. Genes younger than clade 8 are excluded due to low sample sizes.

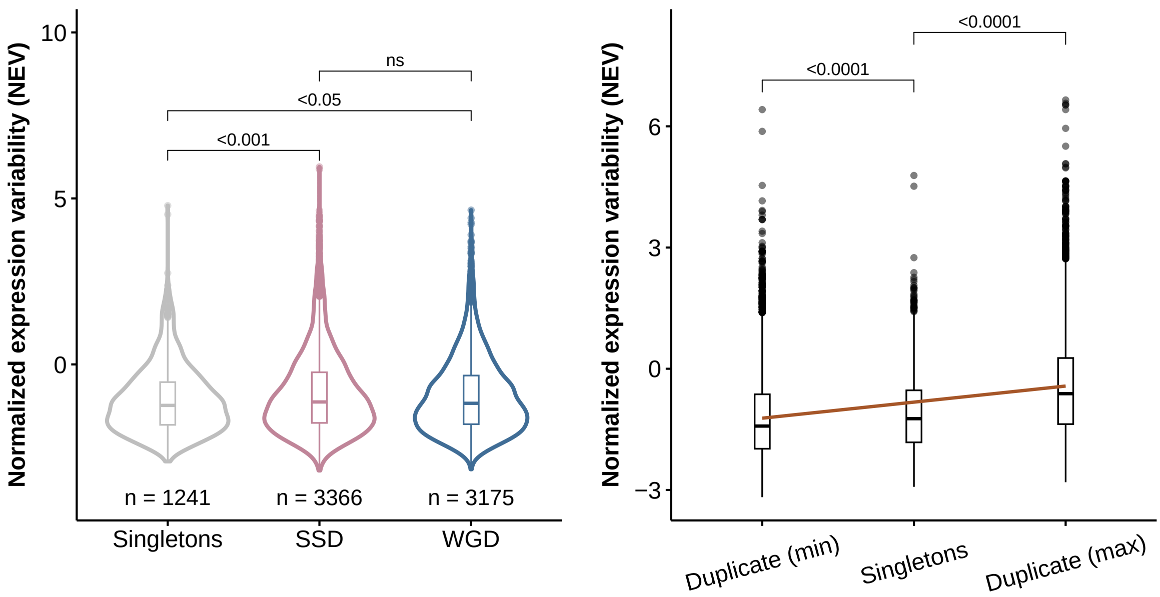

Figure S3: Left: Boxplot mean comparisons of NEV grouped by singletons, genes arising from SSD, and from WGD. Genes born from whole genome duplications (WGDs) and small-scale duplications (SSDs) exhibit higher NEV than singletons (Wilcoxon two-sided test adjusted P = 0.047 between singletons and WGDs, Wilcoxon test adjusted P = 0.00071 between singletons and SSDs, Wilcoxon test adjusted p = 0.27 between WGDs and SSDs. Right: Boxplot mean comparisons of NEV grouped by genes with lower NEVs within a paralog pair, singletons, and genes with higher NEVs within a paralog pair.

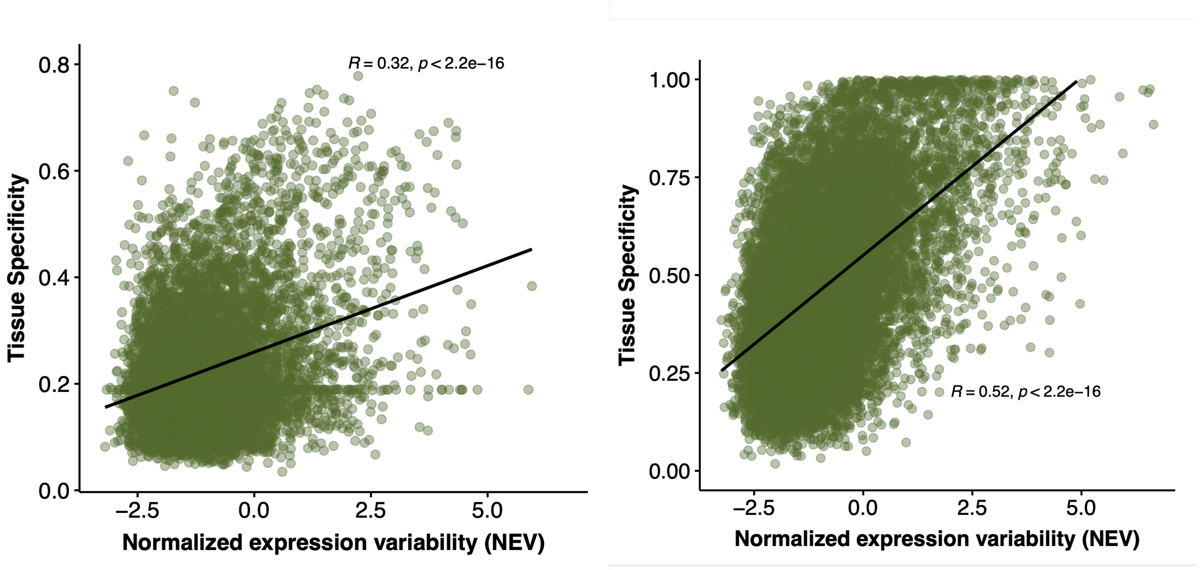

Figure S4: Scatter plots between tissue specificity and normalized expression variability (NEV) using two independent datasets: left from Yang and Gaut 2011, right from Roberts and Josephs 2023.

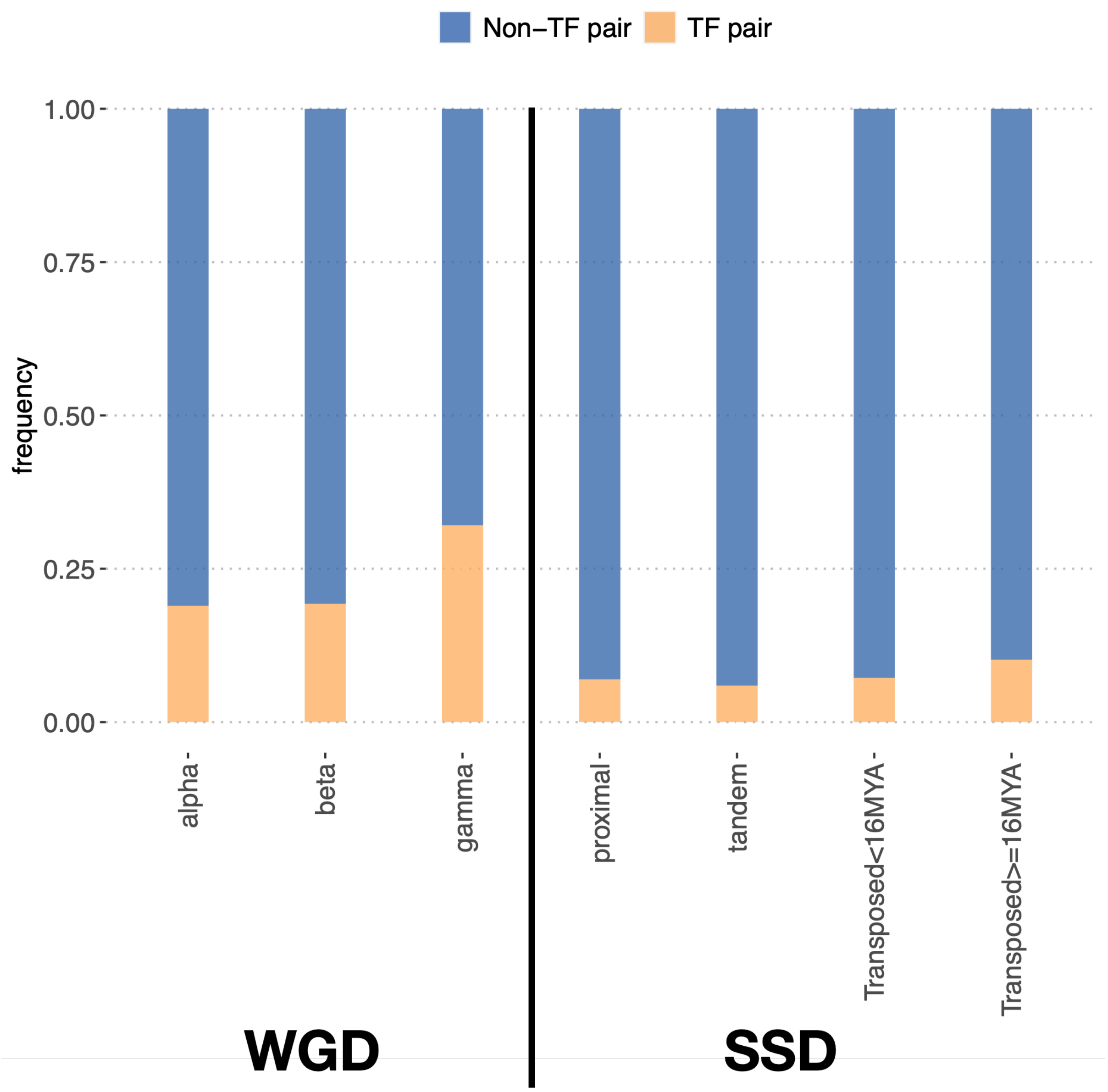

Figure S5: Proportion of non-TF and TF pairs within three whole genome duplications (WGDs) and each subtype of small-scale duplications (SSDs). Paralogs that contain both non-TF and TF genes are excluded. p-value = 5.61e-96 (chi-squared test, WGDs versus SSD)

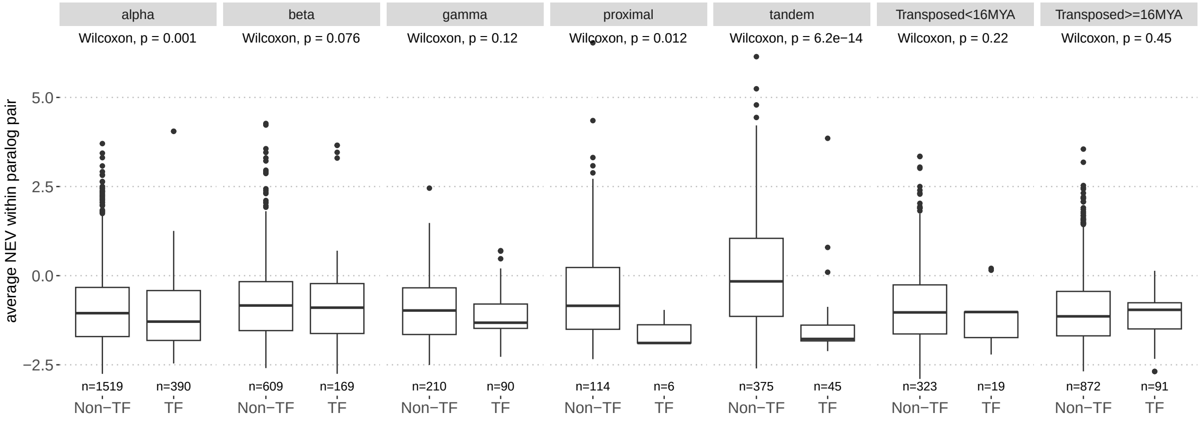

Figure S6: Average normalized expression variability (NEV) within each paralog pair for three whole genome duplications (WGDs) and each sub-types of small-scale duplications (SSDs), grouped by TF or non-TF pairs. Numbers in the plot indicate Wilcoxon test p-values

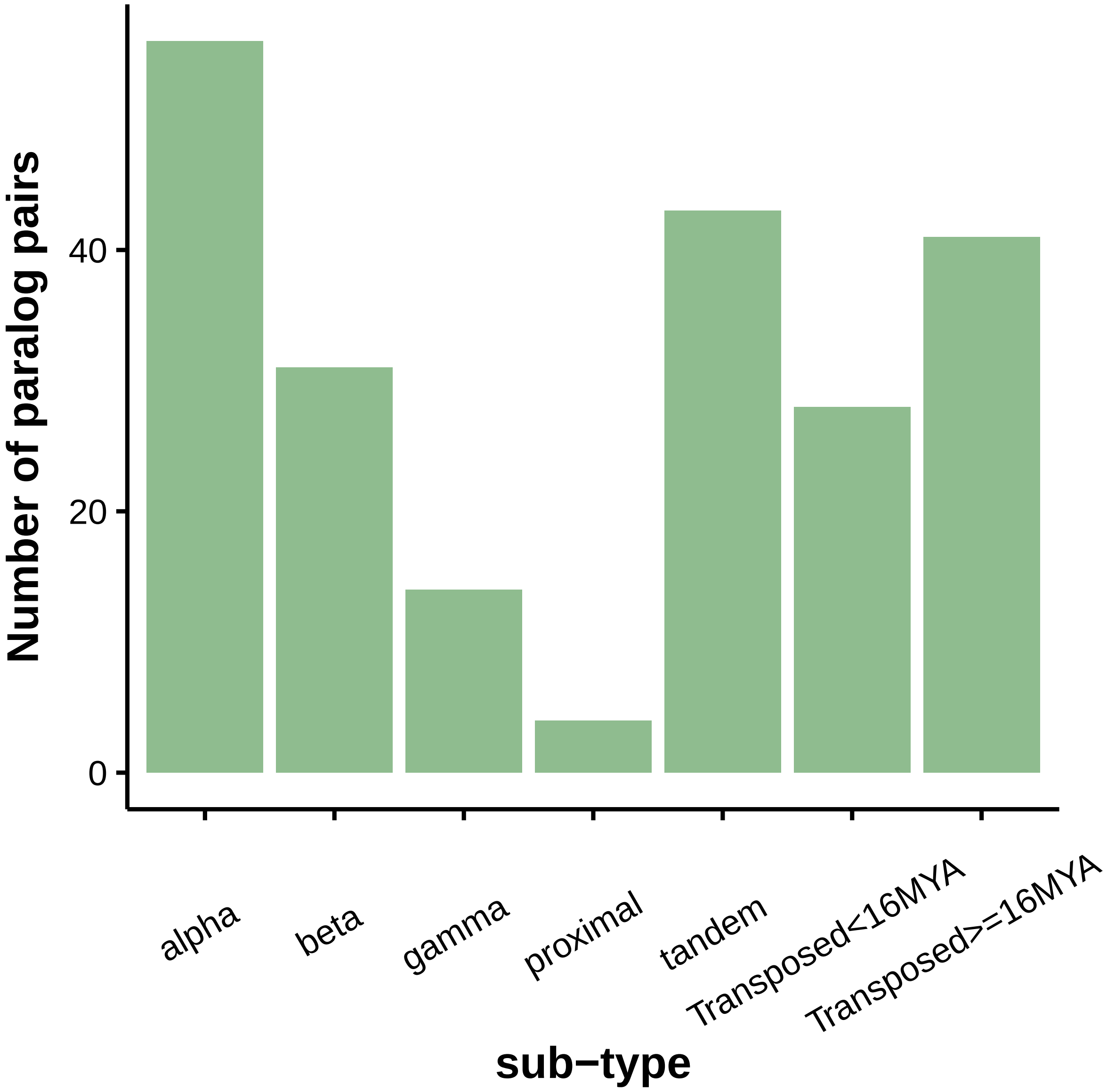

Figure S7: Number of paralog pairs derived from each sub-types of small-scale duplications (SSDs), and three major ancient whole genome duplications (WGDs) for GO enrichment analyses (highest 5% normalized expression variability (NEV) divergence).

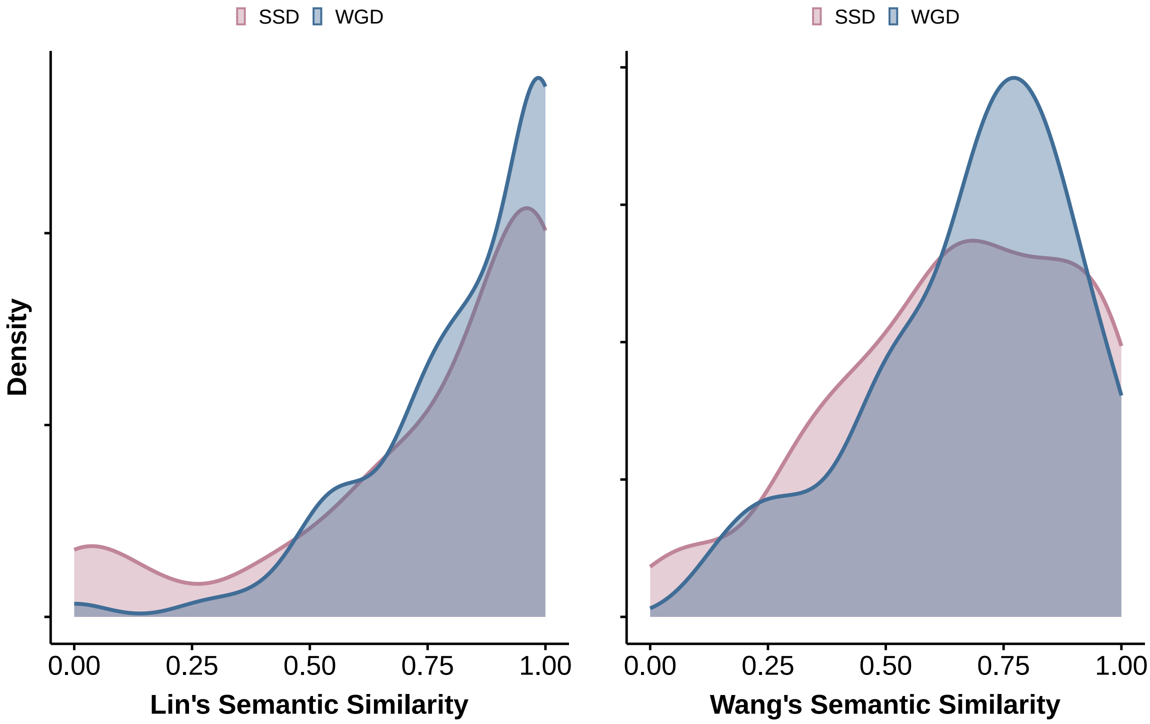

Figure S8: Two additional measures for pairwise semantic analyses in Fig. 2B. R package GOSemSim (Yu et al. 2010) is used with 'Lin' and 'Wang' measures.

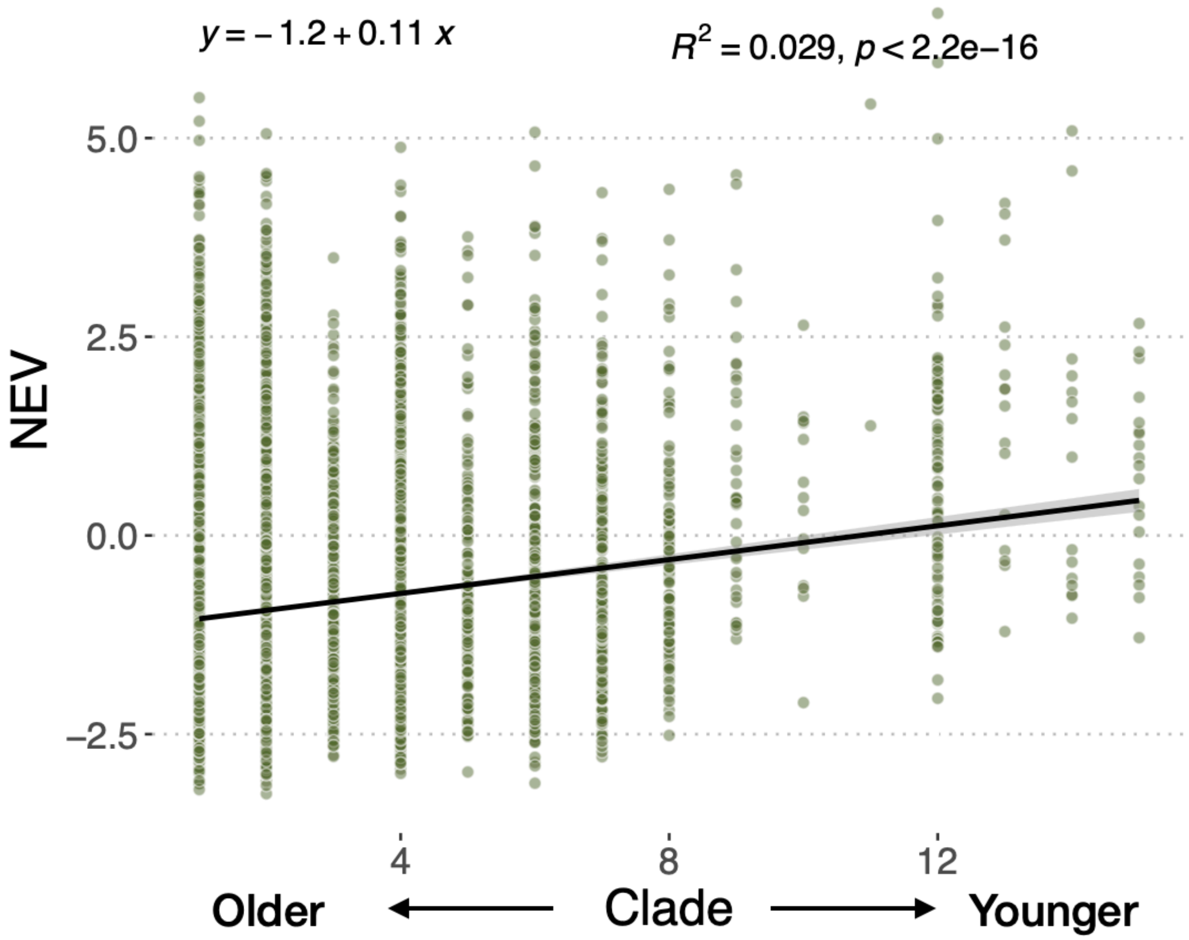

Figure S9: Overall trend between gene age (larger clade represents younger gene) and normalized expression variability (NEV). Higher values of clades represent younger genes. Gene age data were obtained from published data sets (Arendsee et al. 2014).

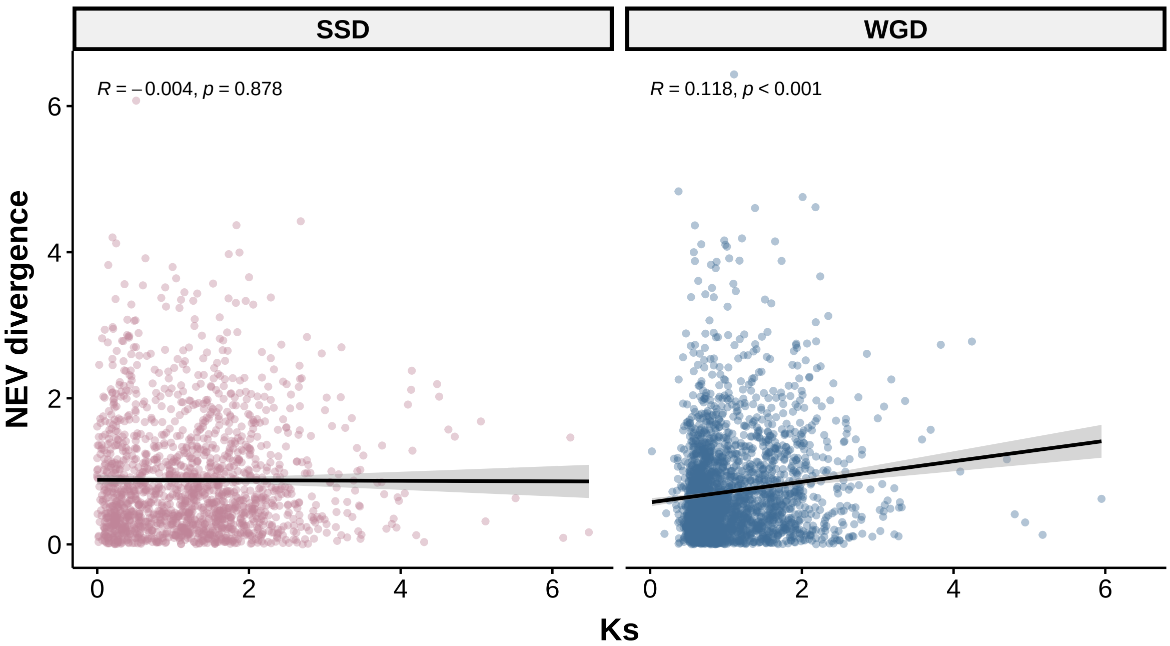

Figure S10: Time-dependent NEV divergence exhibits distinct patterns between SSD and WGD pairs. Scatter plot between NEV divergence and K_s for paralog pairs arising due to SSD and WGD, respectively. The number of synonymous substitutions between aligned protein-coding sequences (K_s) serves as a proxy for duplication age (Ganko et al. 2007). Each dot represents a paralog pair. The black lines are the fitted linear regressions for each plot. Since linear association does not fully capture the relationship between NEV divergence and K_s, we conduct non-linear association analyses using Maximal information coefficient (MIC) (Reshef et al. 2011). The MIC method allows for nonparametric exploration based on maximum information without imposing a strict linear constraint. The p-value of MIC index for WGDs is 0.002, indicating a significant association between K_s and NEV divergence whereas P-value is 0.247 for SSDs. Furthermore, saturation of K_s can occur over extended periods. To ensure robustness, we performed the same analysis for WGDs without gamma duplication events (the oldest WGD event among three major events) (Fig. S12).

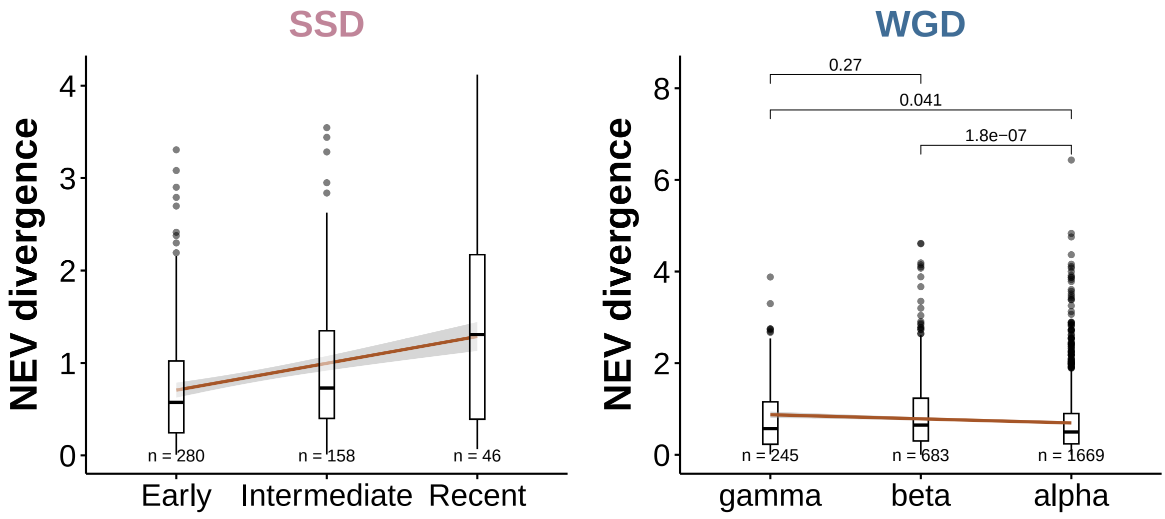

Figure S11: NEV divergence for each paralog pair grouped by estimated time of duplication for SSDs and grouped by known duplication events for WGDs. For SSDs, the time of duplication is approximated by the maximum clade between two paralogs; Gene age data were obtained from published data sets (Arendsee et al. 2014), comprising 17,732 Arabidopsis genes stratified into 15 clades, numbered from the oldest (stratum 1) to the most recent (stratum 15). For this analysis, we first used the maximum clade number within each paralog pair as a proxy for duplication time and then binned paralog pairs into three groups based on this number: early (strata 1 - 2), intermediate (strata 3 - 4), recent (strata 5 - 15). Paralog pairs are not divided with equal intervals in the mean clade number mainly due to unbalanced sample sizes in each clade. For WGD, we use the inferred age of WGD events from oldest (gamma) to the most recent (alpha).

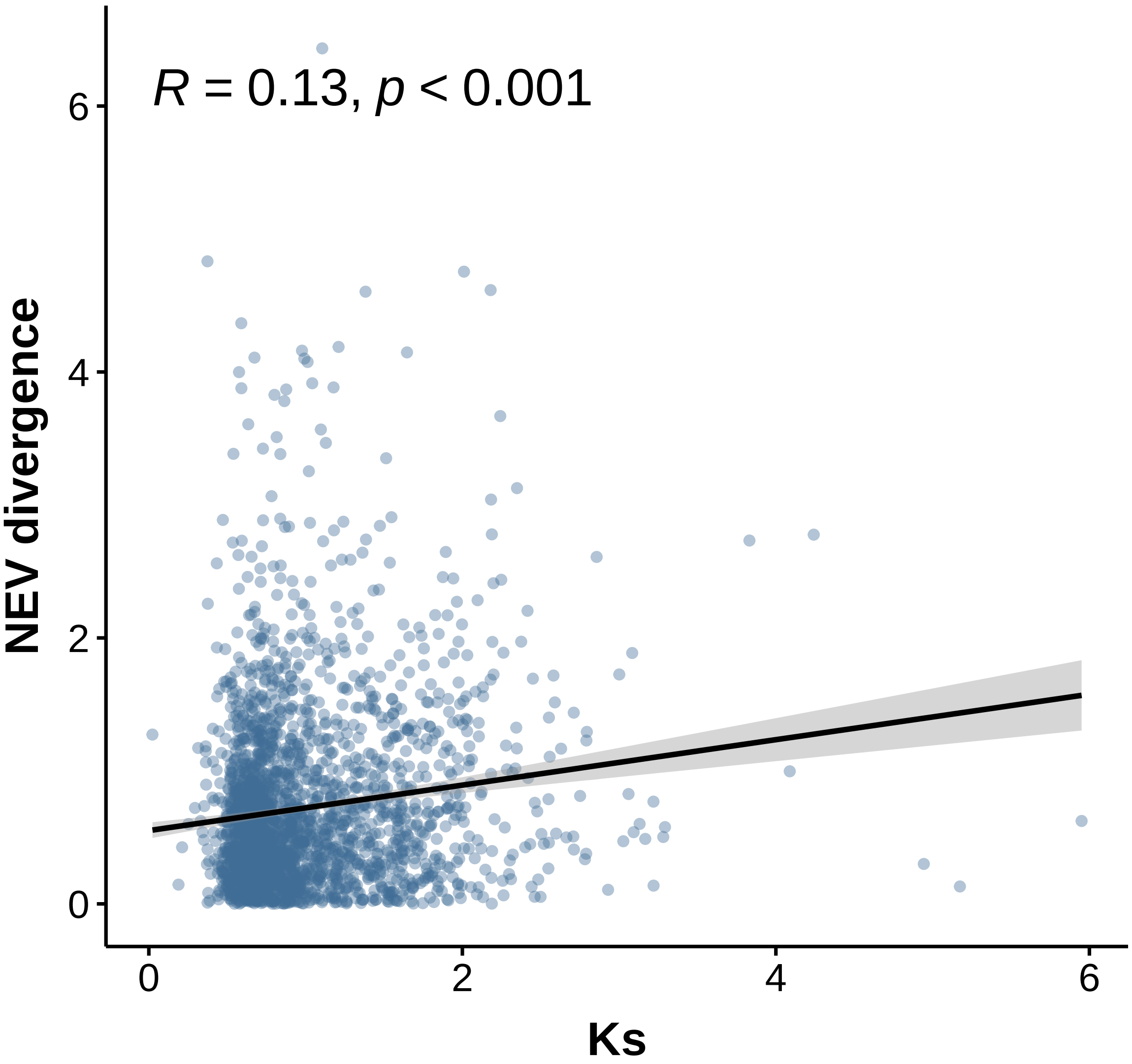

Figure S12: Scatter plot between NEV divergence and K_s for paralog pairs arising from WGD excluding paralogs from gamma duplication event.

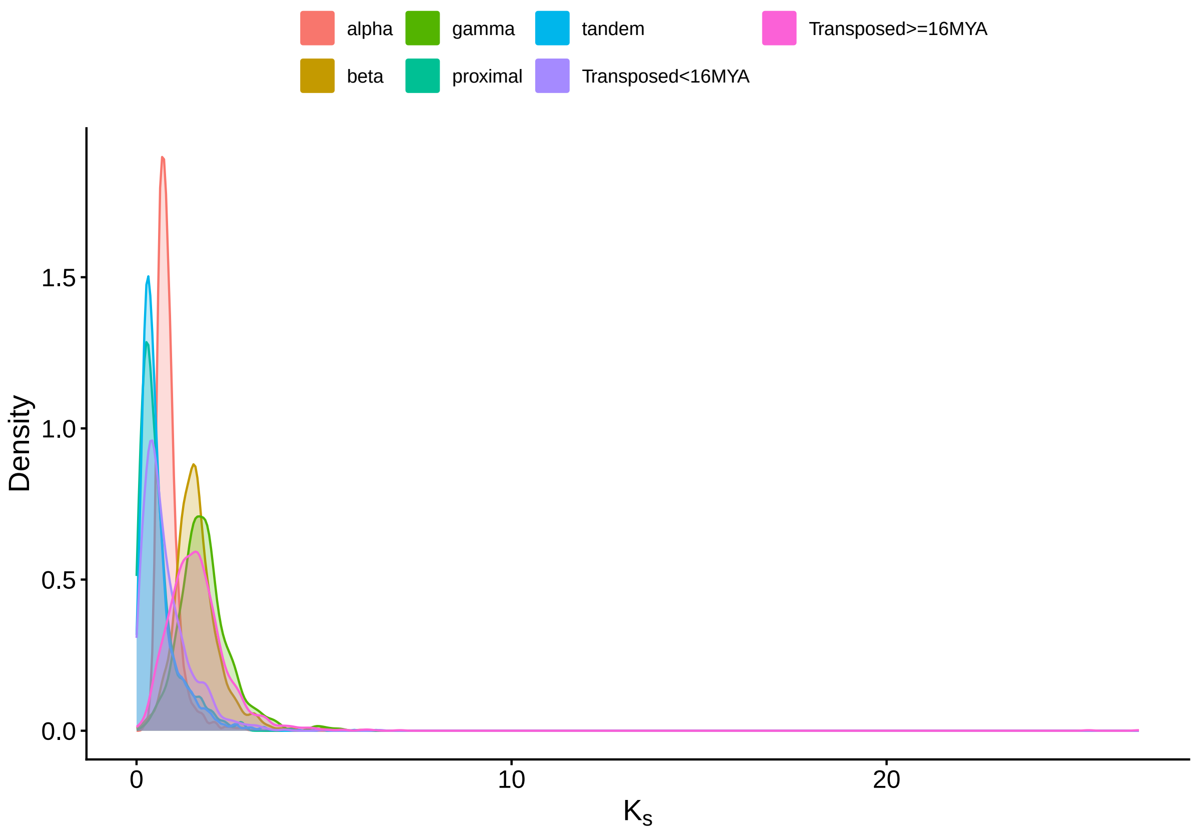

Figure S13: Distribution of K_s values for different duplication types. SSDs (tandem, proximal, and transposed duplications) exhibit lower K_s values than WGDs, indicating that extant small-scale duplicates are typically younger than WGD-derived paralogs in *Arabidopsis*.

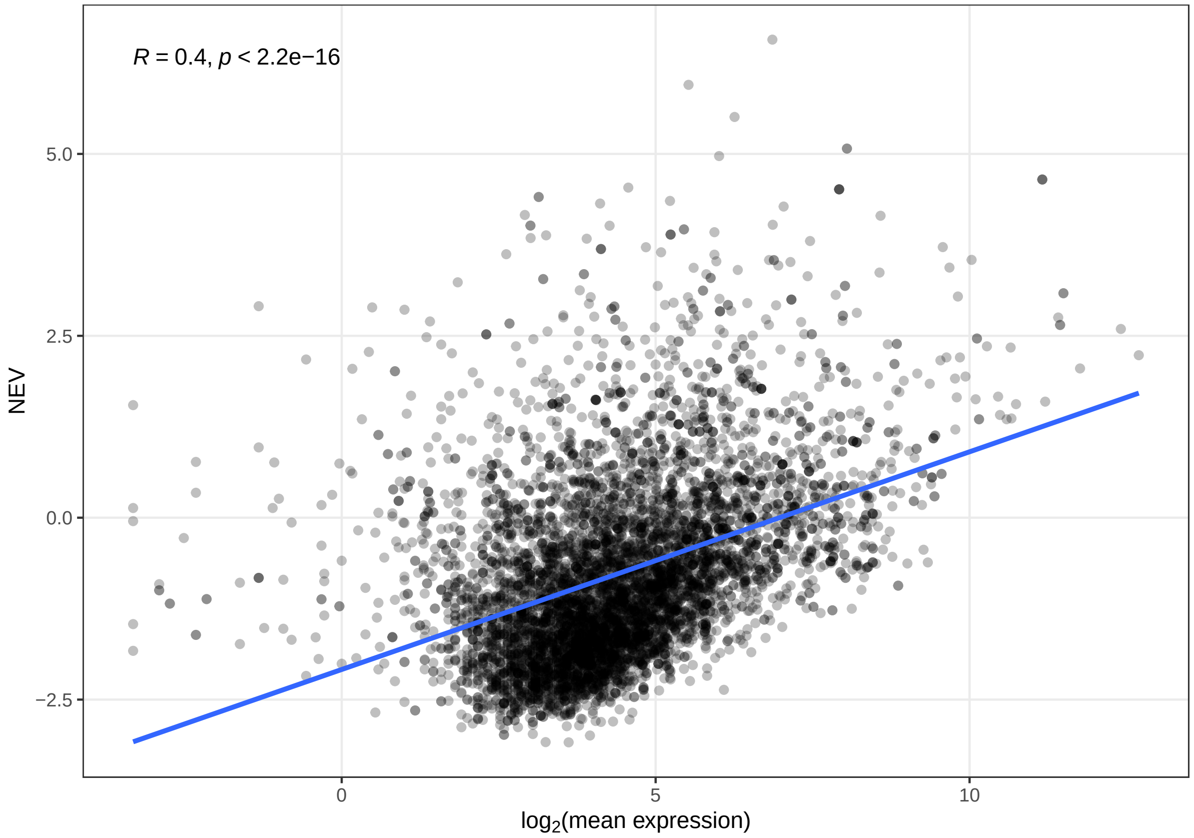

Figure S14: Relationship between gene expression level and normalized expression variability (NEV).

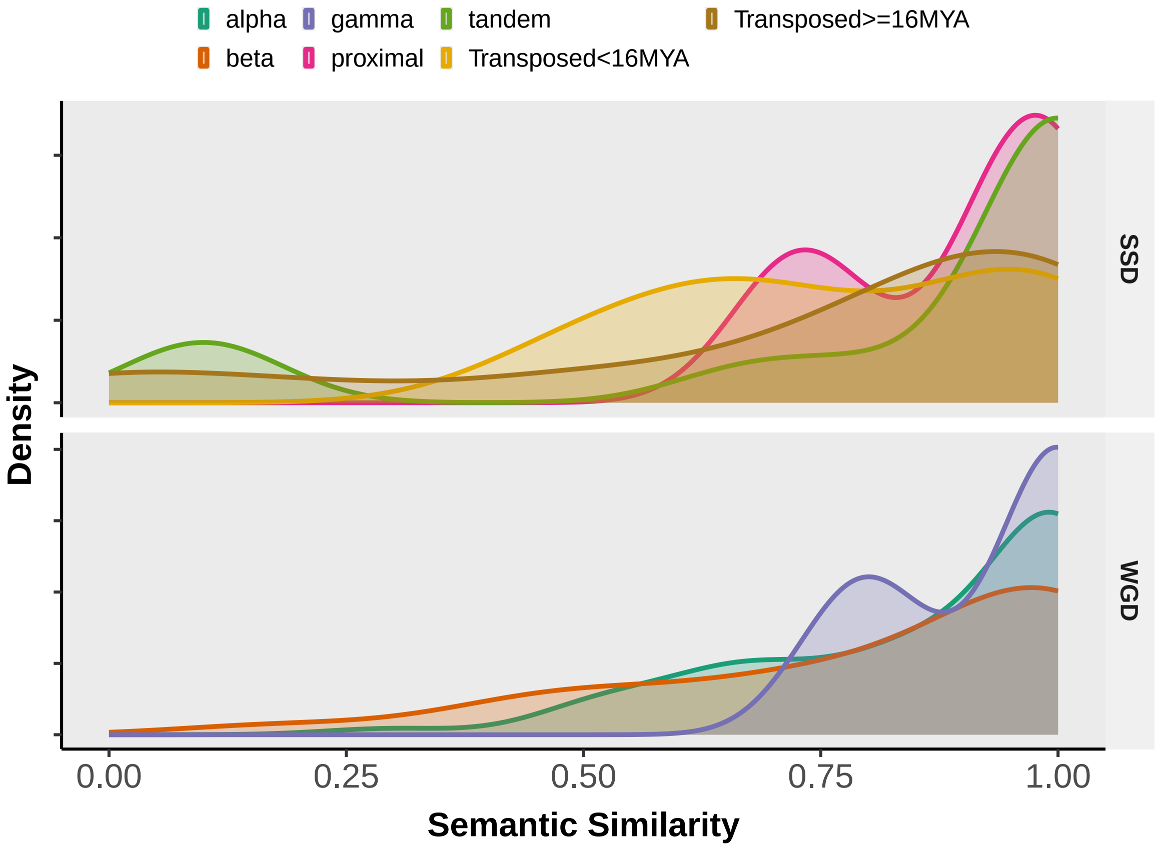

Figure S15: Pairwise semantic similarity analyses within each sub-type of duplications. GO semantic similarity is compared among sub-types of SSDs (tandem, proximal, transposed) and WGDs (alpha, beta, gamma).

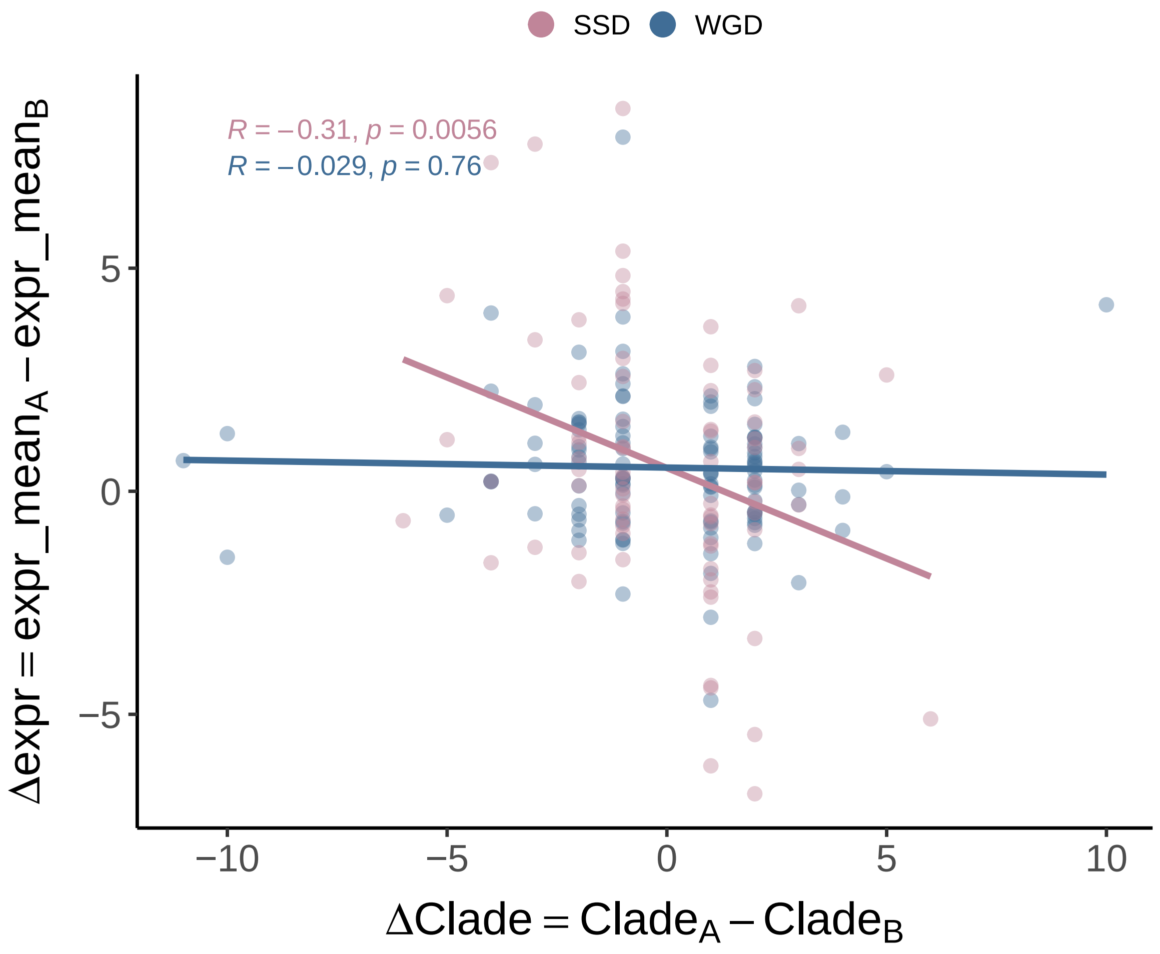

Figure S16: Same analysis as Fig. 3B but replacing NEV with gene expression level. The patterns observed for SSDs here are opposite to those in Fig. 3B.
